# Reovirus uses macropinocytosis-mediated entry and fast axonal transport to infect neurons

**DOI:** 10.1371/journal.ppat.1008380

**Published:** 2020-02-28

**Authors:** Pavithra Aravamudhan, Krishnan Raghunathan, Jennifer Konopka-Anstadt, Amrita Pathak, Danica M. Sutherland, Bruce D. Carter, Terence S. Dermody

**Affiliations:** 1 Department of Pediatrics, University of Pittsburgh School of Medicine, Pittsburgh, Pennsylvania, United States of America; 2 Center for Microbial Pathogenesis, UPMC Children’s Hospital of Pittsburgh, Pittsburgh, Pennsylvania, United States of America; 3 Department of Pediatrics, Vanderbilt University School of Medicine, Nashville, Tennessee, United States of America; 4 Department of Biochemistry and Vanderbilt Brain Institute, Vanderbilt University School of Medicine, Nashville, Tennessee, United States of America; 5 Department of Microbiology and Molecular Genetics, University of Pittsburgh School of Medicine, Pittsburgh, Pennsylvania, United States of America; Indiana University Bloomington, UNITED STATES

## Abstract

Several barriers protect the central nervous system (CNS) from pathogen invasion. Yet viral infections of the CNS are common and often debilitating. Understanding how neurotropic viruses co-opt host machinery to overcome challenges to neuronal entry and transmission is important to combat these infections. Neurotropic reovirus disseminates through neural routes and invades the CNS to cause lethal encephalitis in newborn animals. To define mechanisms of reovirus neuronal entry and directional transport, we used primary neuron cultures, which reproduce *in vivo* infection patterns displayed by different reovirus serotypes. Treatment of neurons with small-molecule inhibitors of different endocytic uptake pathways allowed us to discover that the cellular machinery mediating macropinocytosis is required for reovirus neuronal entry. This mechanism of reovirus entry differs from clathrin-mediated endocytosis, which is used by reovirus to invade non-neuronal cells. Analysis of reovirus transport and release from isolated soma or axonal termini of neurons cultivated in microfluidic devices indicates that reovirus is capable of retrograde but only limited anterograde neuronal transmission. The dynamics of retrograde reovirus movement are consistent with fast axonal transport coordinated by dynein along microtubules. Further analysis of viral transport revealed that multiple virions are transported together in axons within non-acidified vesicles. Reovirus-containing vesicles acidify after reaching the soma, where disassembly of virions and release of the viral core into the cytoplasm initiates replication. These results define mechanisms of reovirus neuronal entry and transport and establish a foundation to identify common host factors used by neuroinvasive viruses. Furthermore, our findings emphasize consideration of cell type-specific entry mechanisms in the tailored design of neurotropic viruses as tracers, oncolytic agents, and delivery vectors.

## Author summary

Viral infections of the central nervous system (CNS) cause a significant health burden globally and compel a better mechanistic understanding of neural invasion by viruses to develop effective interventions. Neurotropic reovirus disseminates through neural routes to infect the CNS and serves as a tractable model to study neural invasion by viruses. Despite knowledge of reovirus neurotropism for decades, mechanisms mediating reovirus neuronal infection remain undefined. We used primary neurons cultured in microfluidic devices to study entry and directional transport of reovirus. We discovered that reovirus uses macropinocytosis for neuronal entry as opposed to the use of a clathrin-mediated pathway in non-neuronal cells. We are unaware of another virus using macropinocytosis to enter neurons. Following internalization, reovirus spreads in the retrograde direction using dynein-mediated fast axonal transport but exhibits limited anterograde spread. We further demonstrate that reovirus disassembly and replication occur in the neuronal soma subsequent to axonal transport. Remarkably, these entry and transport mechanisms mirror those used by misfolded proteins implicated in neurodegenerative diseases. Our findings establish the mechanics of reovirus neuronal uptake and spread and provide clues about therapeutic targets to limit neuropathology inflicted by pathogens and misfolded proteins.

## Introduction

Entry into host cells imposes the first and formidable challenge to viral infection. Cell type-specific distribution of host entry factors dictates species and tissue tropism and, in turn, the pathogenesis of many viruses [1–4]. Polarized cells impose additional restrictions to infection due to asymmetric distribution of receptors and components of the endocytic machinery within the same cell [4–6]. The highly polarized structure of neurons adds a further challenge of overcoming large distances between the soma and the axonal termini that can extend meters in some mammals. Neurotropic viruses internalized at axonal termini require additional mechanisms for efficient navigation through axons to access sites of replication and for neural spread [7–9]. Therefore, challenges to virus entry are cell type-specific and understanding entry mechanisms employed by viruses in the context of cell types implicated in pathogenesis is critical for the development of interventions that block early steps in infection.

Mammalian orthoreovirus (reovirus) is nonenveloped with two concentric protein shells that encapsidate a segmented double-stranded RNA genome [10]. It has a broad host range [11–13], and infection results in age-restricted disease [14, 15]. While reovirus rarely produces serious disease outcomes in humans, reovirus infection may lead to loss of oral tolerance to dietary antigens [16]. Reovirus exhibits serotype-dependent patterns of dissemination and tissue tropism within the central nervous system (CNS). Following a fecal-oral route of transmission, serotype 1 (T1) reovirus spreads primarily through hematogenous routes to infect ependyma [17, 18], while serotype 3 (T3) reovirus spreads by both hematogenous and neural routes to infect CNS neurons [17–19]. Following intramuscular inoculation of mice, both T1 and T3 reovirus infect peripheral motor and sensory neurons, but only T3 efficiently infects CNS neurons to cause neural injury and lethal encephalitis in newborn animals [18, 20]. This serotype-dependent neurotropism genetically segregates with a single domain in the reovirus attachment protein, σ1 [17, 18, 21], indicating that cell-entry mediated by σ1 dictates reovirus neurotropism.

Mechanisms of reovirus entry into transformed and non-polarized cells have been characterized. The σ1 protein mediates reovirus attachment to cells through low-affinity interactions with sialylated glycans followed by higher affinity engagement of proteinaceous receptors that enable virus internalization [22]. Two known receptors allow reovirus cell entry: junctional adhesion molecule A (JAM-A) [23] and Nogo-66 receptor 1 (NgR1) [24]. All reovirus serotypes engage JAM-A [25], which mediates endocytosis in a clathrin-dependent manner [26–28]. The mechanism of reovirus endocytosis following binding to NgR1 is not known. Following receptor-mediated endocytosis, reovirus relies on microtubules and dynein-1 [29, 30] to traverse the endocytic pathway through early, late, and recycling endosomes [31]. Acid-dependent activity of cathepsin proteases in late endosomes uncoats the outer capsid and converts virions into infectious subvirion particles (ISVPs) [32, 33]. Further disassembly of ISVPs aids in penetration of the endosomal membrane and results in the release of transcriptionally active reovirus cores into the cytoplasm [10, 33, 34]. Translation of viral proteins leads to the formation of viral replication organelles, where progeny are assembled for release [10].

In the case of neurons, much of the cell biology of reovirus infection including viral entry is poorly defined. Both sialic acid [35, 36] and JAM-A [35] are dispensable for reovirus neurotropism in mice. Although NgR1 is primarily expressed in neural tissue [37] and is required for reovirus infection of cultured primary neurons [24], the function of NgR1 *in vivo* is not known. Given the differences in receptor engagement, the mechanism of reovirus entry in neurons may differ from non-neuronal cells. Following internalization at nerve termini in peripheral tissues, reovirus can move through neural routes to reach distal sites of replication in the CNS. For instance, following intestinal infection, reovirus enters neurons innervating the myenteric plexus for spread to the CNS [19]. Similarly, following inoculation of hind limb muscle in mice, reovirus directly invades the sciatic nerve to reach spinal neurons [20, 38]. Efficient transport within axons by many neurotropic viruses, including herpesvirus, poliovirus, and rabies virus, requires use of the axonal motors, kinesin and dynein [8, 39, 40]. These motors transport cargo in a highly directional fashion along the polarized microtubule tracks in axons. Kinesin travels anterograde, moving cargo away from the soma, and dynein travels retrograde, moving cargo from axonal termini towards the soma [41]. Although prior *in vivo* studies have implicated fast axonal transport in neural transmission of reovirus [18], the nature of transport has not been directly demonstrated, and the host machinery required for reovirus neuronal transport is not known. Thus, aspects of reovirus neuronal entry including mechanisms of endocytosis and transport and the spatial context of disassembly remain undefined.

In this study, we used primary rat cortical and sensory neuron cultures in combination with microfluidic devices and small molecule inhibitors of endocytosis to determine mechanisms of reovirus neuronal entry and transport. We discovered that reovirus endocytosis in neurons differs from that in other cell types in the use of macropinocytosis as opposed to clathrin-mediated endocytosis (CME). Using microfluidic devices to study directional transport of the virus, we found that reovirus spreads retrograde but not anterograde in cultured neurons. By further tracking single reovirus particles in axons, we determined that the dynamics of motion are consistent with fast transport and mediated by dynein in the retrograde direction. Reovirus axonal transport occurs primarily in non-acidic vesicles, and these vesicles acidify after reaching the soma to allow virion disassembly and entry into the cytoplasm. These results provide comprehensive insights into neuron-specific mechanisms used by reovirus for entry and establish the basis for further exploration of common host factors co-opted by pathogens for neural invasion.

## Results

### Serotype-dependent neuronal infection by reovirus is reproduced using primary neuron cultures

To establish *in vitro* systems to study serotype-specific mechanisms of reovirus neuronal entry and transport, we cultivated primary neurons from embryonic rats. Cortical neurons (CNs) and dorsal root ganglion neurons (DRGNs) were chosen to represent neuronal tissue infected by reovirus in the central and peripheral nervous system, respectively. Reovirus strains T1L and T3SA+ were chosen to represent the two serotypes with differing CNS tropisms [17, 22]. These strains share all gene segments except S1, which encodes the σ1 attachment protein.

**Fig 1:**
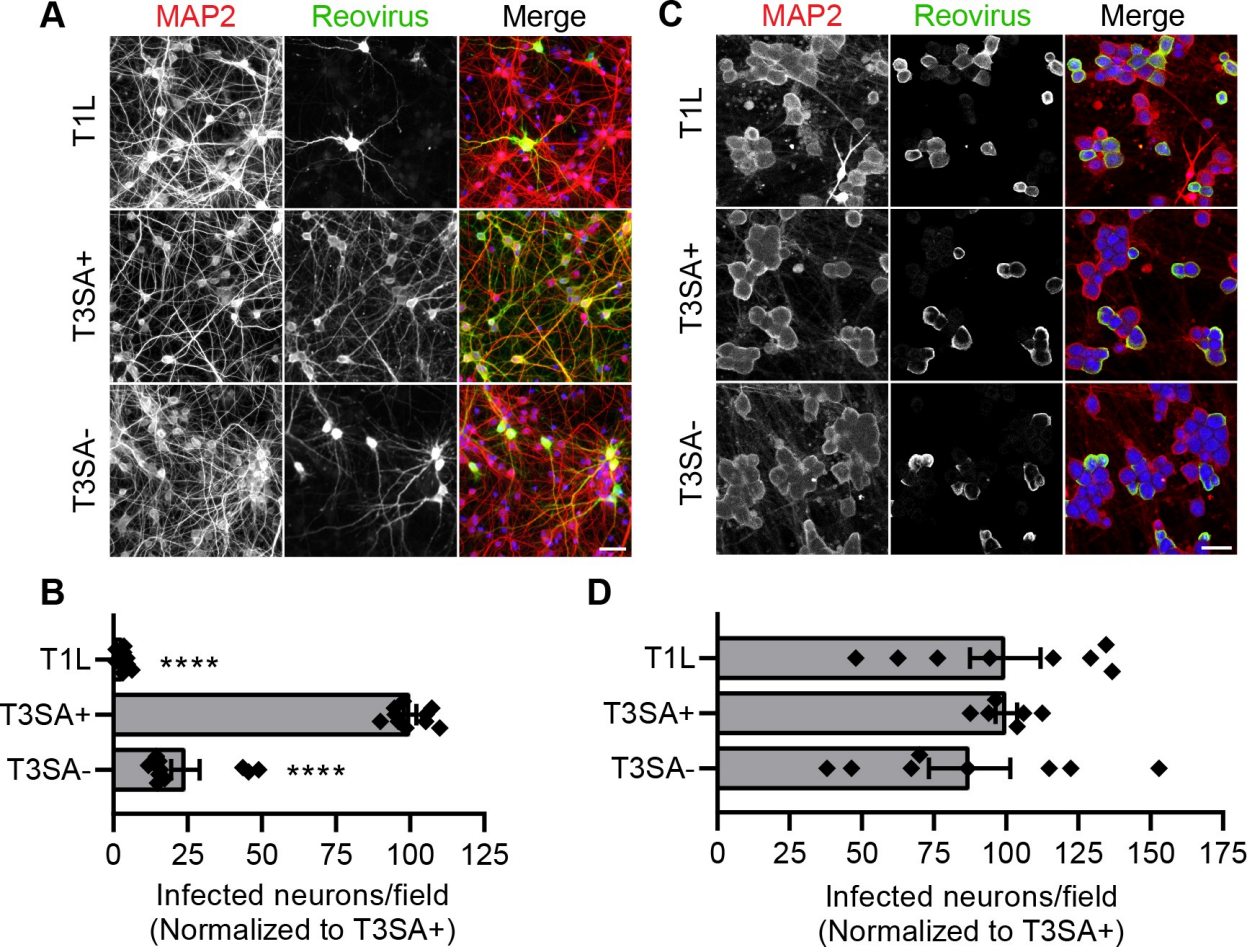
Serotype-dependent reovirus infection *in vivo* is reproduced in primary neuron cultures. Cultured rat cortical neurons (CNs) or dorsal root ganglion neurons (DRGNs) were adsorbed at an MOI of 500 or 50 PFU per cell, respectively, with the reovirus strains shown, and cells were fixed at 24 h post-adsorption. (A, C) Neurons were immunostained using an antibody to detect a neuronal protein (MAP2) and reovirus-specific antiserum. Nuclei were stained with DAPI (blue). Representative micrographs display infected CNs (A) or DRGNs (C). Scale bars, 50 μm. (B, D) Infectivity of CNs (B) or DRGNs (D) was scored as the mean number of cells stained by reovirus antiserum per field-of-view. Bars indicate means from at least three independent experiments, each with duplicate or triplicate samples, normalized to T3SA+ infectivity. For reference, ~ 26% of CNs are infected by T3SA+ reovirus under the experimental conditions used.) Error bars indicate SEM. Individual data points are normalized averages from 8 to 12 fields-of-view from each sample. Values that differ significantly from T3SA+ by ANOVA and Dunnett's test are indicated (****, *P* < 0.0001).

To test whether serotype-dependent infection by reovirus is reproduced in cultivated neurons, CNs and DRGNs were adsorbed with T1L or T3SA+ and infectivity was determined 24 h post-adsorption by enumerating reovirus-antigen-positive cells. T1L infectivity of DRGNs was comparable to that of T3SA+. In contrast, T1L infected CNs poorly when compared with T3SA+ (**Fig 1A–1D**). We also tested the requirement of sialic acid engagement in reovirus infection of different neuron types using strain T3SA-. The σ1 protein of T3SA- contains a single polymorphism (P204L) that abrogates its binding to sialic acid [22]. While T3SA- infected CNs significantly less efficiently than did T3SA+, both strains infected DRGNs comparably (**Fig 1A–1D**). These data are consistent with previous reports of CN infectivity by reovirus [21, 42] and further suggest that reovirus infection of DRGs is not dependent on the serotype or the capacity to bind sialic acid. Overall, the observed infectivity of cultured neurons mirrors serotype-dependent infection *in vivo* [20, 42] and validates the use of CN and DRGN cultures to study mechanisms of reovirus entry and transport in neurons. These results also indicate that, of the strains tested, T3SA+ maximally infects both neuron types used in this study.

### Reovirus spreads predominantly in a retrograde direction in DRGNs

**Fig 2:**
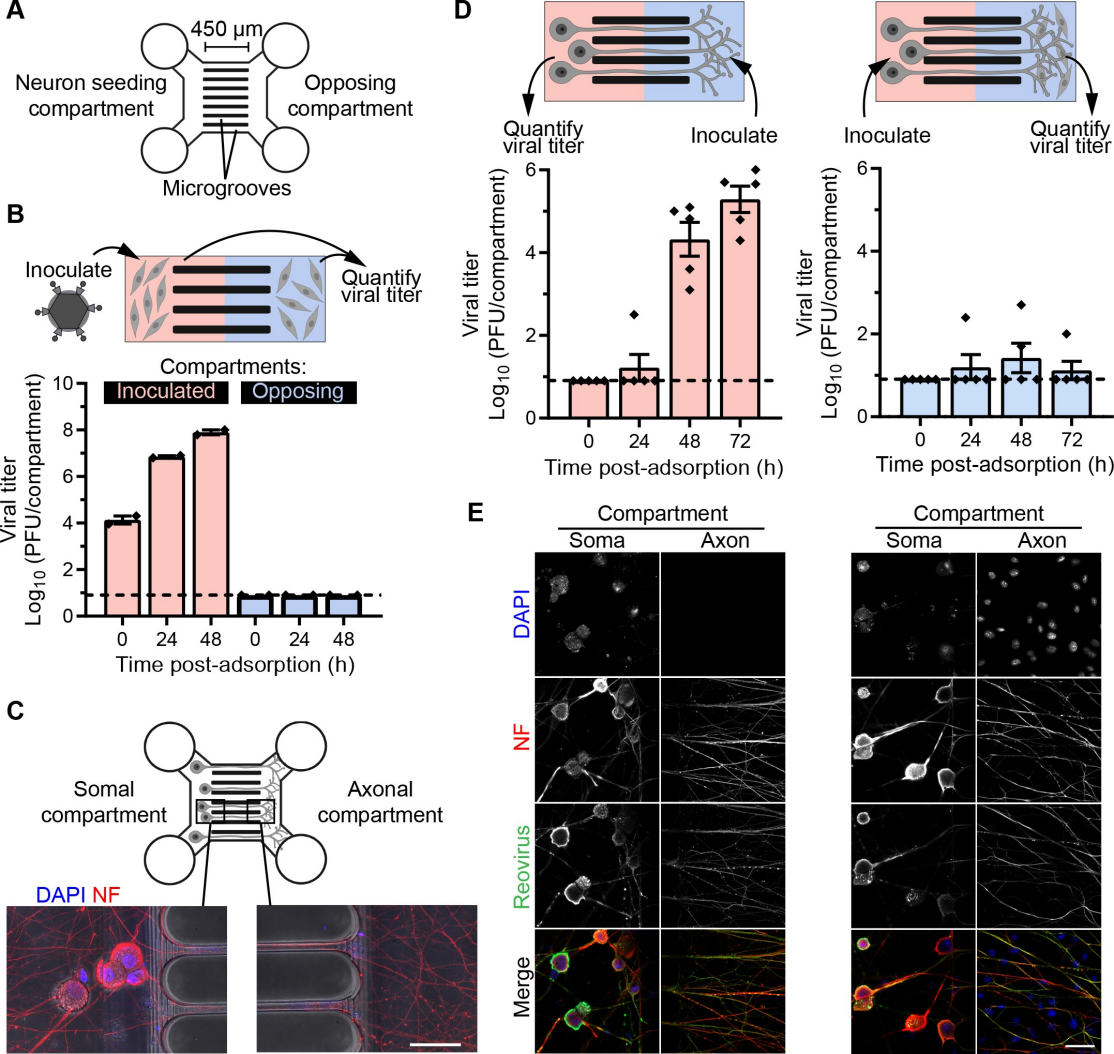
Anterograde spread of reovirus is limited in DRGNs. (A) Schematic of a microfluidic device with two compartments connected by 450-μm-long microgrooves. (B) L929 cells were cultivated in both compartments (red and blue) of the microfluidic device. Cells in the left compartment were adsorbed with T3SA+ virions at an MOI of 10 PFU/cell, and viral titers in culture supernatants from both inoculated and opposing compartments were determined at the indicated times post-adsorption. Bars indicate mean titers of duplicate devices from one representative experiment. Error bars indicate SEM. (C) Representative micrographs show somal and axonal compartments of a microfluidic device with DRGNs cultivated for 7 days and stained with markers for nuclei (DAPI) and axons (non-phosphorylated neurofilament H, NF). (D-E) DRGNs cultivated in microfluidic devices were adsorbed with T3SA+ virions in the somal or axonal compartment as indicated in the schematics (D, top). L929 cells were cultivated in the axonal compartment to amplify virus released by anterograde spread following inoculation of the somal compartment. Viral titers in the culture supernatant in the compartment opposing the inoculated compartment at the indicated times post-adsorption are shown (D). Bars indicate means, and error bars indicate SEM. Individual data points represent titers from five devices in total from three independent experiments. Devices were fixed at 72 h post-adsorption and immunostained with reovirus antiserum and a neuronal marker (NF). Representative micrographs of somal and axonal compartments corresponding to the experimental setup in (D) are shown in (E). Scale bars, 50 μm. In B and D, dashed lines mark the limit of detection.

To investigate directional transport of reovirus in neurons, we used microfluidic devices that allow isolation and differential treatments of neuronal soma and axons. These devices consist of two compartments connected by thin, micron-sized grooves (~ 3 μm diameter, 450 μm long; **Fig 2A**). The compartments are fluidically isolated by the dimensions of the grooves and hydrostatic pressure introduced by maintaining a higher volume of medium in one compartment relative to the other [43]. To confirm fluidic isolation in the microfluidic device, L929 cells, which are highly susceptible to reovirus infection, were cultivated in both compartments of the device. Cells in the left compartment were adsorbed with T3SA+ virions, and a higher volume of medium was maintained in the opposing compartment (**Fig 2B, schematic**). Culture supernatants were collected from both compartments at 24 h intervals, and viral titers were determined. While titers in the inoculated compartment increased over time (**Fig 2B**), no titer was detected in the opposing compartment, thus demonstrating efficient fluidic isolation of the compartments.

Next, we used the microfluidic device to study directional spread of reovirus in neurons. DRGNs plated in the left compartment of the microfluidic device, designated as the somal compartment, extended axons through the microgrooves into the opposing compartment, designated as the axonal compartment, and formed an elaborate axonal network within 7 days (**Fig 2C**). However, CNs plated in these devices did not maintain viability and, therefore, were not used in these studies. To determine whether reovirus can spread bidirectionally, DRGNs cultivated in microfluidic devices were adsorbed either in the somal or the axonal compartment with T3SA+ virions. Culture supernatants from both compartments were collected at 24 h intervals, and viral titers were determined by plaque assay. Reovirus titer could be recovered from the inoculated compartment at all time points examined (**S1 Fig**). Under fluidic isolation, transport through axons is required for the virus to reach and be released from neuronal extensions in the opposing compartment. Following inoculation of the axonal compartment, soma were infected, and viral titer was recovered from the somal compartment by 48 h post-adsorption (**Fig 2D and 2E**), suggesting that reovirus is capable of retrograde spread. Following inoculation of the somal compartment, no appreciable titer was detected in supernatants from the axonal compartment, even after 72 h of incubation (data not shown). It is possible that the amount of virus released following anterograde spread was below the limit of detection. To amplify reovirus that may be released, L929 cells were cultivated in the axonal compartment. However, following inoculation of the somal compartment, L929 cells were rarely infected, judging from the presence of viral antigen, and little titer was detected in supernatants from the axonal compartment (**Fig 2D and 2E**), indicating that reovirus spreads inefficiently in the anterograde direction. This limited spread is not due to the absence of entry or infection of soma, as most neurons in the somal compartment were infected (**Fig 2E**). The infected soma include those proximal to microgrooves, which likely extend axons into the opposing compartment. These results indicate that while reovirus efficiently spreads retrograde in DRGNs, anterograde spread is limited.

### Retrograde neuronal transport of reovirus is fast and mediated by dynein

**Fig 3:**
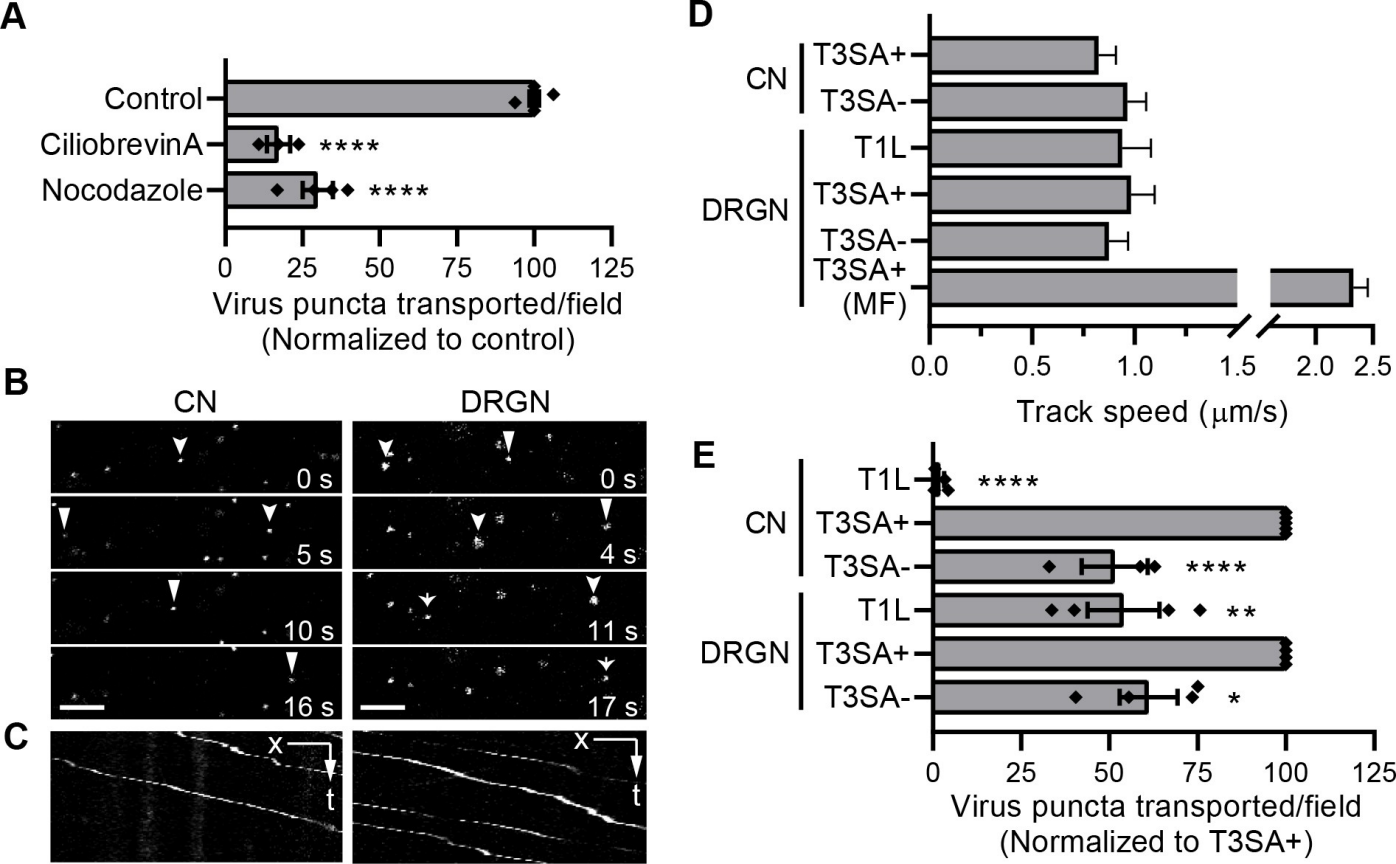
Reovirus undergoes microtubule-dependent fast axonal transport in neurons. (A) CNs were treated with the drugs shown, adsorbed with T3SA+ virions, and viral puncta transported per field-of-view were enumerated following treatment. Bars indicate mean values from at least two independent experiments, each with one to two samples, normalized to untreated control. Error bars indicate SEM. Individual data points are averages from 8 to 12 fields-of-view from each sample. Values that differ significantly from control by ANOVA and Dunnett's test are indicated (****, *P* < 0.0001). (B-E) CNs or DRGs were adsorbed with fluorescently-labeled T1L, T3SA+, or T3SA- virions for 30 min. The inoculum was removed, and the motion of virions was recorded using live-cell fluorescence imaging. (B) Time-lapse images of representative fields are shown for T3SA+ with positions of individual puncta tracked over time with discrete arrows or arrowheads. (C) Kymographs corresponding to B show the positions (x) of individual puncta along an axon over time (t). Scale bars, 5 μm. (D) Track speed was calculated for the reovirus strains shown as the overall distance traveled by each fluorescent reovirus puncta during the tracked interval (*n* > 300 tracks). MF refers to neurons cultured in microfluidic devices. Bars indicate medians, and error bars indicate 95% confidence intervals. (E) Fluorescent puncta transported for distances greater than 5 μm in each 82 x 82 μm field-of-view during 1 min of live-cell imaging were enumerated. Bars indicate means normalized to T3SA+, and error bars indicate SEM. Individual data points are averages from 8 to 12 fields-of-view for each sample. Values that differ significantly from T3SA+ by ANOVA and Dunnett's test are indicated (*, *P* < 0.05; **, *P* < 0.01; ****, *P* < 0.0001).

Many neurotropic viruses engage dynein motors for fast, microtubule-dependent retrograde transport [8, 39, 44, 45]. To test whether microtubule-dependent fast transport is required for reovirus movement, CNs were treated with nocodazole to disrupt microtubules or ciliobrevin A to inhibit dynein [46] and adsorbed with fluorescently-labeled T3SA+ virions. The motion of fluorescently-labeled reovirus puncta in randomly selected ~ 82 x 82 μm areas covered with neurites was imaged over 1 min intervals (**S2A Fig; S1 Movie**). Most puncta in each field were stationary, uninternalized virions, likely either bound to cell-surface receptors or adhered non-specifically to the culture dish (**S2A Fig; S1 Movie**). Reovirus puncta that travelled distances greater than 5 μm in each field during the imaging interval were enumerated under each condition. Disrupting microtubules or inhibiting dynein significantly diminished transport of T3SA+ in CNs (**Fig 3A**), suggesting that reovirus transport in neurons is dependent on microtubules and mediated by dynein.

To define reovirus transport characteristics, the motion of fluorescently-labeled T3SA+ virions 45 min post-adsorption of CNs and DRGNs cultivated in glass-bottomed dishes was analyzed. In these experiments using dense neuron cultures, the direction of transport (anterograde vs retrograde) cannot be ascertained. However, we assume that the transport was predominantly in the retrograde direction based on two observations: (i) inhibiting the retrograde transport motor, dynein, significantly diminishes reovirus transport in CNs and (ii) reovirus is transported predominantly in the retrograde direction, with little reversal of direction in axons of DRGNs cultivated in microfluidic devices (**S2 Movie**). Therefore, puncta that traveled > 6.5 μm within the imaging interval (1 min) were considered to be in retrograde motion and further analyzed for motion kinetics. T3SA+ puncta were transported with a median speed of ~ 0.9 μm/s in both CNs and DRGNs (**Fig 3B–3D**).

To compare serotype- and strain-dependent transport dynamics, we also analyzed the motion of T1L or T3SA- virions. Both of T1 and T3 reovirus moved over long distances (> 6.5 μm) in each field-of-view in DRGNs, whereas only T3 reovirus strains were transported in CNs (**Fig 3E**). Interestingly, the capacity of reovirus to traffic in different neuron types correlates with infectivity (**Figs 1B, 1D** and **3E**), further confirming that the initial steps in reovirus entry mediated by σ1 dictate the capacity of reovirus to infect specific neuron types. T1 virions were transported at a median speed of ~ 0.9 μm/s in DRGNs (**Fig 3D**), which is comparable to the transport speed of both T3SA+ and T3SA- reovirus (~ 0.9 μm/s) in both CNs and DRGNs (**Fig 3D; S2B Fig**). These velocities also are comparable to dynein-mediated fast transport of neuronal cargo and other viruses [8, 40, 47, 48]. The dynamics of T1L in CNs could not be assessed, as there was no detectable transport of this strain. In both CNs and DRGNs, all reovirus strains paused during transport, spending ~ 17% of the time paused and ~ 3.6 s in each pause (**S2C and S2D Fig**). The comparable transport dynamics of T3 reovirus strains (T3SA+ and T3SA-) in CNs and DRGNs suggests use of similar transport machinery regardless of the neuron type and the capacity of the strain to bind sialic acid. Furthermore, comparable transport dynamics of T1 and T3 reovirus strains in DRGNs suggest that both reovirus serotypes share similar transport machinery following endocytosis.

We further compared the transport characteristics of T3SA+ virions in DRGNs cultivated in microfluidic devices versus dishes. Surprisingly, virions were transported with a significantly greater instantaneous and average velocity (~ 2.3 μm/s) in microfluidic devices relative to transport in dishes (**Fig 3D; S2B Fig**). Virions also rarely paused during transport but spent a longer duration (~ 5.7 s) in each paused state in microfluidic devices relative to the transport behavior in dishes (**S2C and S2D Fig**). These results are likely attributable to differences in transport dynamics in regions of axons proximal and distal to the soma [41, 49], which cannot be distinguished while imaging dense neuron cultures in dishes, whereas only axons distal to soma are imaged within the grooves in microfluidic devices.

Multiple regulatory factors coordinate retrograde axonal transport of cargo. Many neurotropic viruses rely on these factors and some are even capable of inducing their translation for transport [9]. To test the requirement for new protein synthesis in reovirus transport, CNs were treated with cycloheximide to inhibit protein synthesis, and reovirus transport was quantified following the treatment. Cycloheximide treatment did not affect the number of reovirus puncta transported, suggesting that new protein synthesis is not required for reovirus neuronal transport (**S3A Fig**). However, cycloheximide treatment effectivity abrogated new viral protein synthesis, as concluded from the lack of staining of viral antigens 24 h post-adsorption of T3SA+ (**S3B Fig**). These results demonstrate that molecular factors required for reovirus neuronal transport are present at steady state and need not be newly synthesized.

### Multiple reovirus particles are transported together in neurites

**Fig 4:**
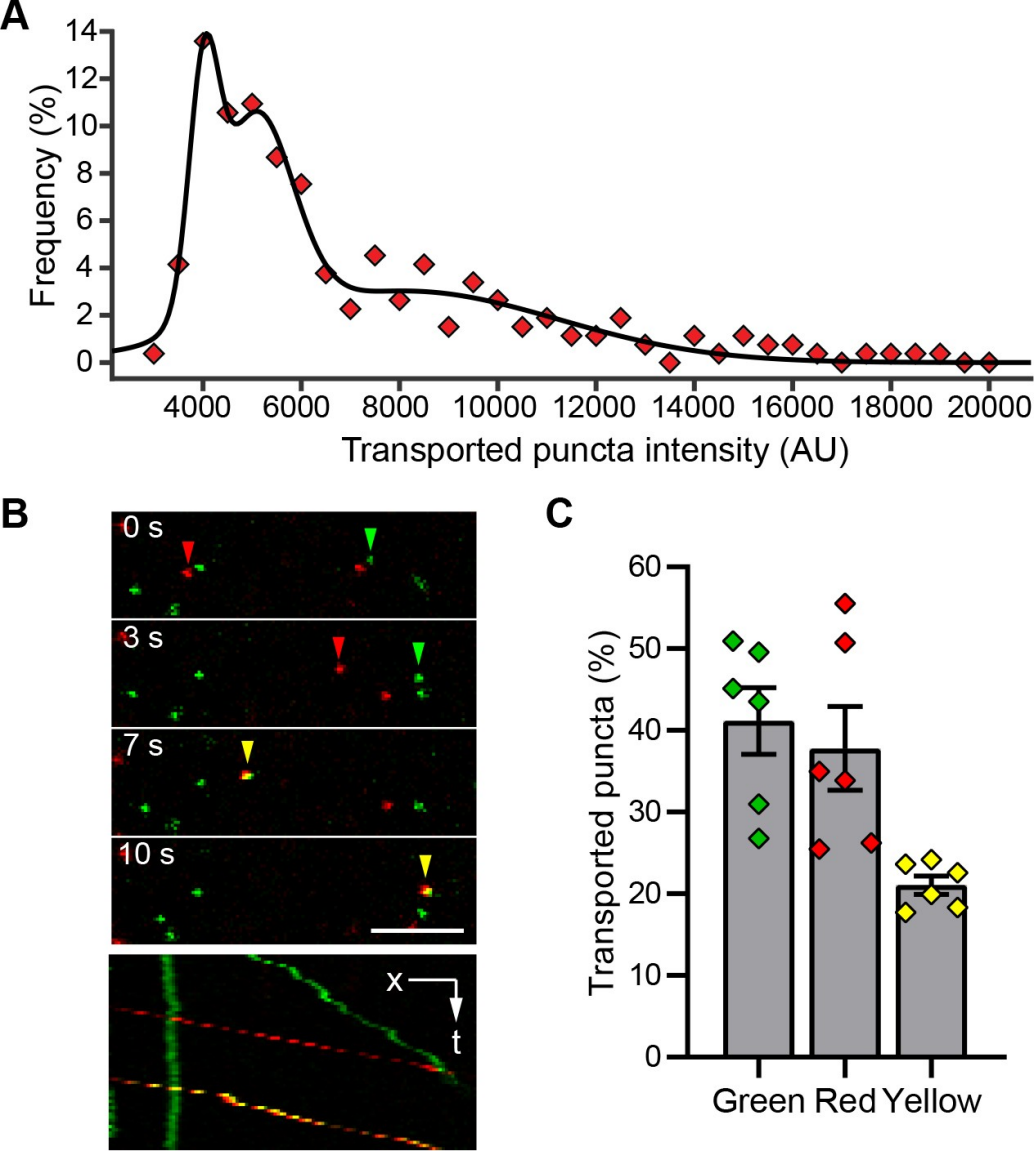
Multiple reovirus particles are transported together in axons. (A) CNs were adsorbed with Alexa Fluor 647-labeled T3SA+ for 45 min. The distribution of intensities of individual transported puncta and a multi-peak Gaussian fit of intensities are shown (*n* = 265 puncta). (B-C) Cortical neurons were adsorbed with an equal number of Alexa Fluor 488- (green) and Alexa Fluor 647-labeled (red) T3SA+ virions. Puncta transported in axons that contain virions labeled with one (green or red) or both (yellow) fluorophores are indicated in representative time-lapse images and the corresponding kymograph (B) and the percentage of each is quantified (C). Scale bar, 5 μm. Bars indicate mean values. Error bars indicate SEM. Data are representative of three independent experiments each with duplicate samples and a total of 1072 puncta. Individual data points are averages from 8 to 12 fields-of-view from each sample.

Reovirus puncta undergoing neuronal transport display a wide range of fluorescence intensities. The intensity distribution fit well with a multiple peak Gaussian curve (**Fig 4A**). If the lowest intensity peak in the distribution corresponds to that of a single particle, then the spread of the distribution suggests that a majority of the transported puncta contain at least two and up to five reovirus particles. To test this possibility, T3SA+ virions were labeled with either Alexa Fluor 488 (green) or Alexa Fluor 647 (red). CNs were adsorbed for 30 min with a 1:1 mixture of particles labeled with each fluorophore before simultaneous time-lapse imaging of both fluorophores. A significant fraction (~ 21%) of transported puncta contained particles labeled with both fluorophores (yellow; **Fig 4B and 4C; S3 Movie**) and the results were consistent between different preparations of purified virions. If we assume that these puncta contain at least two particles, by probability, a further ~ 21% of puncta also would contain two particles labeled with the same fluorophore. Therefore, we estimate that 42% of the transported puncta contain at least two virions. These observations could result from co-endocytosis of multiple particles or fusion of endosomes containing individual particles prior to or during transport.

### Macropinocytosis, and not clathrin-mediated endocytosis, mediates reovirus entry into neurons

**Fig 5:**
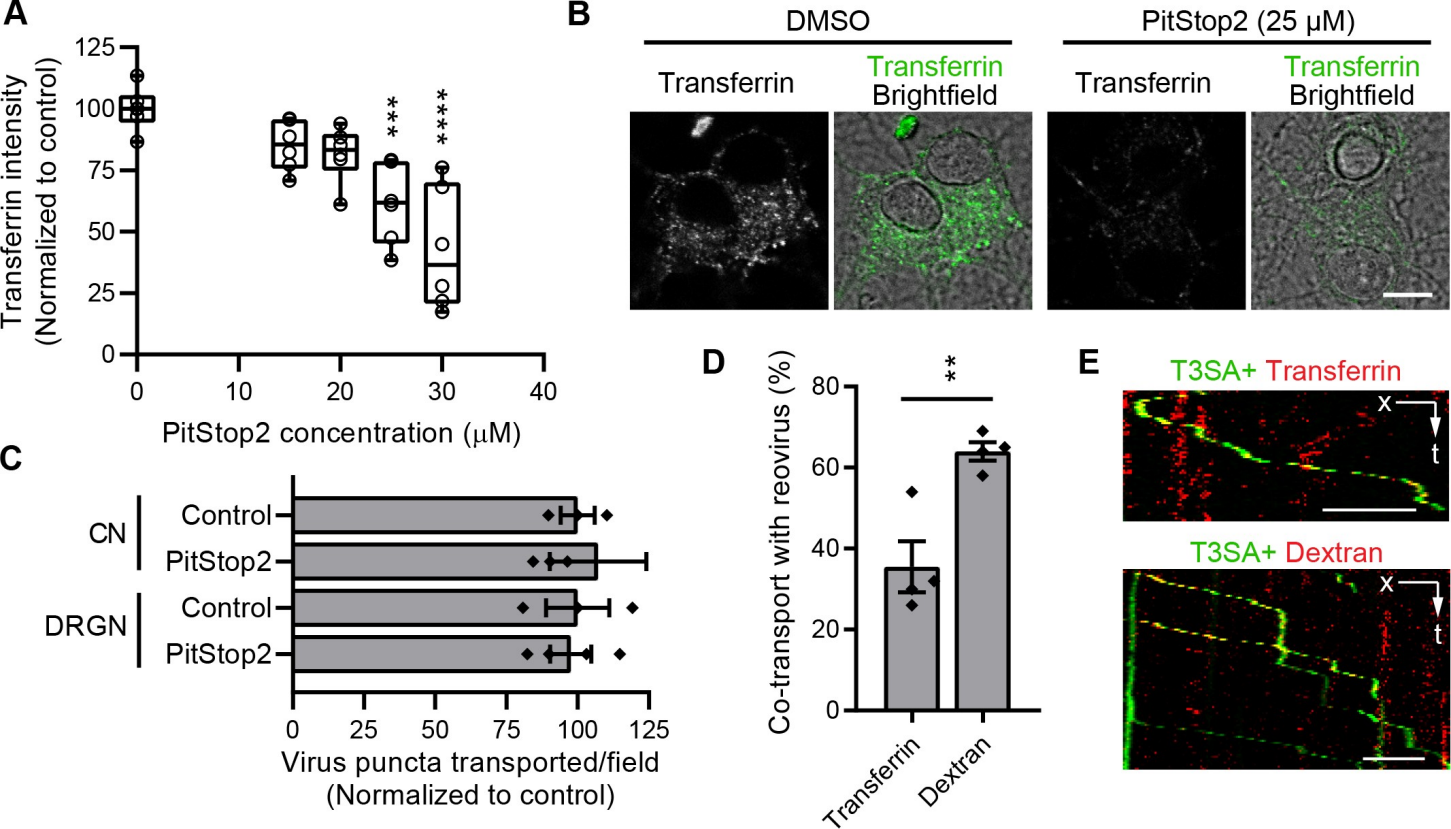
Clathrin-mediated endocytosis is not required for reovirus entry into neurons. (A) CNs were treated with PitStop2 at he concentrations shown, and the uptake of transferrin-Alexa Fluor 488 was quantified following treatment. Data are presented in a box plot with horizontal bars marking 25^th^ and 75^th^ percentiles and median values, and the whiskers marking extreme values. Individual data points represent means normalized to DMSO-treated controls from three independent experiments, each with duplicate samples. Values that differ significantly from control by ANOVA and Dunnett's test are indicated (***, *P* < 0.001; ****, *P* < 0.0001). (B) Representative micrographs show reduced uptake of transferrin following treatment with 25 μm PitStop2 compared with a DMSO-treated control. Scale bar, 10 μm. (C) CNs or DRGNs were treated with 25 μm PitStop2 (Table 1), and T3SA+ puncta transported per field-of-view were enumerated following treatment. Bars indicate means of four samples normalized to DMSO-treated control. Error bars indicate SEM. Individual data points are averages from 8 to 12 fields-of-view per sample. Values did not differ significantly from control as determined by *t*-test (*P >* 0.05). (D-E) CNs were incubated with fluorescently-labeled transferrin or dextran (10 kDa) and T3SA+ virions. The percentage of reovirus puncta that co-traffic with transferrin or dextran is shown (D) along with representative kymographs (E). **, *P* < 0.01 determined by *t*-test. Bars indicate means of duplicate samples from two independent experiments. Error bars indicate SEM. Individual data points represent averages from 8 to 12 fields-of-view from each sample (*n* = 184 and 335 reovirus puncta analyzed for co-transport with transferrin and dextran, respectively). Scale bar, 5 μm.

CME is essential for efficient reovirus entry into multiple cell types [26, 27]. To test the function of CME in reovirus neuronal entry, we used PitStop2, a well-characterized small-molecule inhibitor of CME [50]. PitStop2 treatment inhibited uptake of transferrin, a classical marker for CME [51], into CNs in a dose-dependent manner (**Fig 5A and 5B**). A 25 μM dose reduced transferrin uptake to 60% of control, DMSO-treated cells and was used in further experiments. To quantify the effect of CME inhibition on reovirus uptake, CNs were treated with PitStop2 for 30 min prior to and during adsorption with Alexa Fluor 488-labeled T3SA+ virions. At 45 min post-adsorption, cells were fixed, and extracellular virions were stained under non-permeabilizing conditions with reovirus polyclonal antiserum and a secondary antibody conjugated with Alexa Fluor 647 (**S4A Fig, schematic**). With this dual-staining strategy, internalized particles are labeled with Alexa Fluor 488 alone, while extracellular particles are labeled with both fluorophores (**S4A Fig**). Surprisingly, blocking CME with PitStop2 did not affect reovirus internalization into CNs (**S4B Fig)**. PitStop2 treatment also did not affect the number T3SA+ reovirus puncta transported per field-of-view, another step in reovirus entry, in both CNs and DRGNs (**Fig 5C**). These findings suggest that CME is not required for reovirus entry into neurons.

To further verify that CME is dispensable for reovirus neuronal entry, we analyzed co-transport of reovirus with fluorescently-labeled transferrin and high-molecular-weight-dextran (10 kDa), which is too large for uptake into endocytic vesicles and instead is internalized by macropinocytosis [52, 53]. CNs were incubated with fluorescently-labeled transferrin or dextran and T3SA+ virions for 30 min and then analyzed for co-transport of reovirus puncta with each of the markers. While a fraction of reovirus puncta (~ 35%) was co-transported with transferrin, a significantly higher fraction was transported with dextran (~ 65%, **S4 Movie**), suggesting that reovirus enters neurons predominantly via a clathrin-independent pathway, perhaps macropinocytosis (**Fig 5D and 5E**).

**Fig 6:**
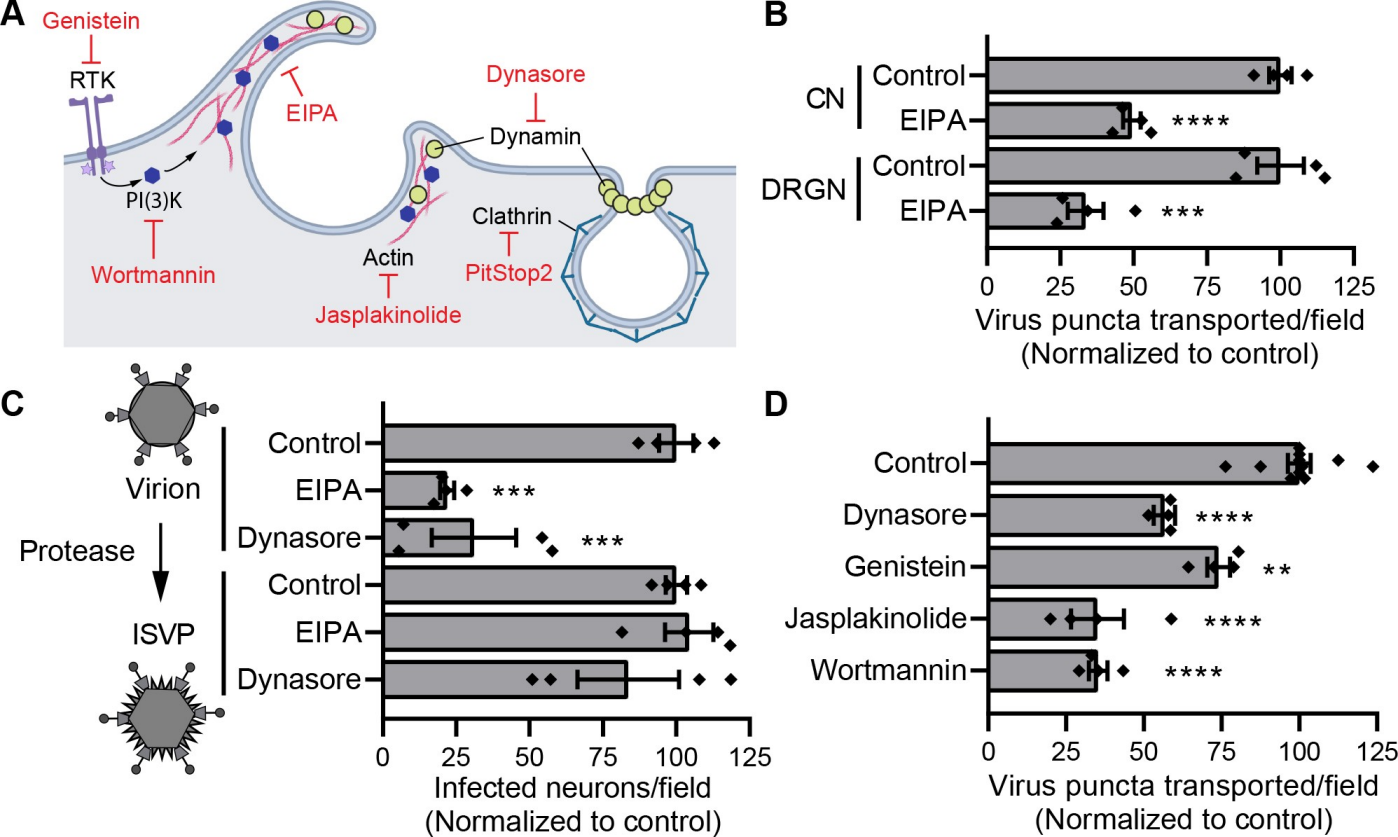
Macropinocytosis-mediated entry is required for reovirus neuronal transport and infection. (A) Schematic shows host factors mediating endocytic pathways and corresponding small molecule inhibitors (red) (RTK, receptor tyrosine kinase). (B, D) CNs and DRGNs (B) or CNs (D) were treated with the drugs shown (Table 1), adsorbed with T3SA+ virions, and viral puncta transported per field-of-view were enumerated following treatment. (C) CNs were treated with the drugs shown (Table 1), adsorbed with T3SA+ virions or ISVPs at an MOI of 50,000 particles/cell, and infectivity was quantified 24 h post-adsorption. In B-D, bars indicate means normalized to untreated controls. Error bars indicate SEM. Data are representative of at least two independent experiments each with one to three samples. Individual data points are averages from 8 to 12 fields-of-view per sample. Values that differ significantly from control by ANOVA and Dunnett's test are indicated (**, *P* < 0.01; ***, *P* < 0.001; ****, *P* < 0.0001).

To test whether macropinocytosis mediates reovirus neuronal entry, we used ethyl isopropyl amiloride (EIPA) as a specific inhibitor of this internalization mechanism [54]. EIPA acts by inhibiting Na+/H+ exchange, which lowers sub-membrane pH and impairs actin assembly and activation of host factors required for the formation of membrane ruffles that initiate macropinocytosis (**Fig 6A**). Short-term treatment with EIPA (1 h) significantly reduced reovirus internalization into CNs (**S4B Fig**) and reovirus transport in both CNs and DRGNs (**Fig 6B**). However, EIPA treatment did not affect uptake of transferrin or cholera toxin subunit B, markers for CME and clathrin-independent endocytosis [55], respectively, confirming that the effects of EIPA on reovirus entry are specific to inhibition of macropinocytosis (**S4C and S4D Fig**). To further test the specific function of macropinocytosis in reovirus entry, we used ISVPs, which are produced *in vitro* by limited-proteolysis of virions [56]. ISVPs recapitulate reovirus disassembly intermediates that can directly penetrate the plasma membrane and bypass the requirement for endocytosis [57] (**Fig 6C, schematic**). EIPA limited infection of CNs by T3SA+ virions but not by ISVPs (**Fig 6C**), demonstrating that virions and not ISVPs specifically require macropinocytosis to enter neurons. Additionally, these results indicate that the drug acts to block early reovirus entry steps leading up to the formation of ISVPs and does not inhibit infection as a consequence of cytotoxicity. The inhibitory effect of EIPA on reovirus infectivity of DRGNs also is observed following infection at a multiplicity of infection (MOI) of 5 PFU/cell, indicating that entry by macropinocytosis is not a consequence of the MOI used to assess infectivity (**S4E Fig**).

Macropinocytosis requires an ensemble of molecular components to generate, extend, and close membrane ruffles and scission them into macropinosomes. Macropinocytosis and related processes are functional in neurons and play crucial roles in remodeling growth cones during development [58, 59] and in retrieving synaptic vesicle membranes following intense neuronal activity [60]. These processes require actin dynamics, phosphoinositide 3-kinase (PI(3)K) signaling, and dynamin, among other factors [60, 61]. To determine the requirement of these factors in reovirus entry through macropinocytosis into CNs, we used small molecule inhibitors to specifically block each factor and assessed the effect on the number of reovirus puncta transported per field-of-view. Short-term treatment with jasplakinolide, which disrupts actin filaments [62] and wortmannin [63] or dynasore [64], which inhibit PI(3)K and dynamin, respectively, significantly reduced transport of reovirus in CNs (**Fig 6D**). Dynasore also significantly reduced infectivity of T3SA+ virions but not ISVPs, further demonstrating a requirement for dynamin in neuronal infection by reovirus virions and the specific effect of the drug on early steps in entry (**Fig 6C**). The effects of jasplakinolide and wortmannin on infectivity could not be assessed, as long-term treatment (24 h) with these drugs compromised neuronal viability (data not shown). However, short-term term treatments (less than 2 h) with these inhibitors did not alter neuronal transport. Therefore, treatments with these inhibitors were limited to < 2 h.

While constitutive in some immune cells, macropinocytosis is regulated in most other cell types and can be triggered by the activation of receptor tyrosine kinases (RTKs) [65–67]. To test whether reovirus can trigger RTKs for neuronal entry, we treated CNs with genistein, a tyrosine kinase inhibitor [68], for 1 h prior to viral adsorption. Genistein treatment significantly reduced the number of reovirus puncta transported in CNs (**Fig 6D**), suggesting that reovirus triggers macropinocytosis by activating RTKs for neuronal entry. Collectively, these results demonstrate that reovirus neuronal entry requires Na+/H+ exchange, actin dynamics, and activities of PI(3)K, dynamin, and tyrosine kinases, consistent with a critical role for macropinocytosis in this process.

### Disassembly of reovirus occurs in the neuronal soma after retrograde transport

**Fig 7:**
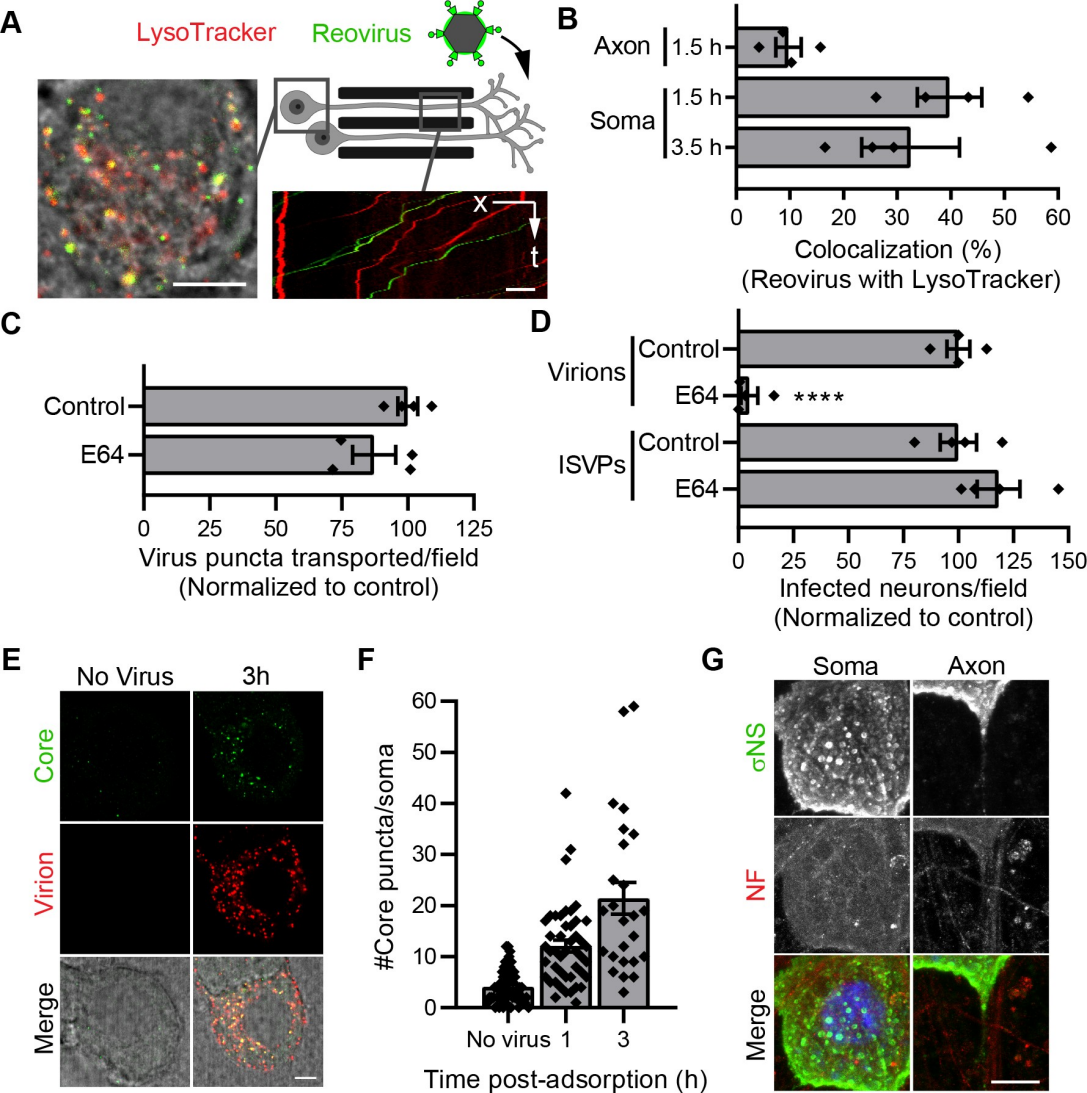
Disassembly of reovirus occurs in neuronal soma following axonal transport. (A-B) DRGNs cultivated in microfluidic devices were adsorbed in the axonal compartment with fluorescently-labeled T3SA+ virions (green) for 1 h. LysoTracker (red) was added to both compartments during the last 20 min of virus adsorption, and cells were imaged at the times shown after virion addition. Representative images show LysoTracker and reovirus distribution in a DRG soma (A—left, micrograph) and motion in axons within a microgroove (A—right, kymograph). Scale bars, 5 μm. The percentage of reovirus puncta co-transported with LysoTracker in axons within the microgrooves or colocalized with LysoTracker in the soma were quantified (B). Bars indicate means from at least two independent experiments with a total of four devices. Error bars indicate SEM. Individual data points are averages from at least 16 microgrooves containing axons or 8 to 12 fields-of-view containing soma from each device. (C) CNs were treated with E64 (Table 1), and T3SA+ puncta transported per field-of-view were enumerated following treatment. (D) CNs were treated with E64, adsorbed with T3SA+ virions or ISVPs at an MOI of 50,000 particles/cell, and infectivity was quantified at 24 h post-adsorption. In C and D, bars indicate means normalized to untreated controls. Error bars indicate SEM. Data are representative of two independent experiments, each with duplicate samples. Individual data points are averages from 8 to 12 fields-of-view from each sample. Values that differ significantly from control by *t*-test are indicated (****, *P* < 0.0001). (E-F) DRGNs were adsorbed with fluorescently-labeled T3SA+ virions (red) for 30 min and incubated at 37°C for 1–3 h before fixation and staining with anti-core polyclonal serum (green). Representative fluorescence micrographs (E) and quantification of the number of reovirus core puncta per soma (F) are shown. Scale bar, 5 μm. Bars indicate means from two samples each with > 23 soma. Error bars indicate SEM. (G) Representative micrographs show distribution of T3SA+ replication factories stained by σNS antiserum and axons stained by an antibody against non-phosphorylated neurofilament H (NF). Scale bar, 10 μm.

Vesicle maturation in the endocytic pathway is accompanied by a decrease in pH, which cues the disassembly of many viruses. Proteolytic processing of reovirus outer-capsid proteins requires cathepsin proteases that are activated by acidic pH in late endosomes [32]. To determine where the reovirus-containing macropinosomes acidify in neurons, we used LysoTracker to mark acidic vesicles. DRGNs cultivated in microfluidic devices were adsorbed with fluorescently-labeled reovirus in the axonal compartment and stained with LysoTracker before imaging reovirus transport in axons within the microgrooves and soma (**Fig 7A, schematic**). Only a minority of virus puncta transported in axons co-stained with LysoTracker (**S5 Movie**) compared with a significantly higher fraction of puncta that colocalized in the soma (**S6 Movie**; **Fig 7A and 7B**). These results indicate that reovirus-containing vesicles acidify after axonal transport in the soma, which likely marks the site of reovirus disassembly.

Fluorophores covalently conjugated to reovirus outer-capsid proteins are removed following proteolytic disassembly [32, 69]. Therefore, it is possible that a fraction of virions, which may be quickly disassembled following vesicle acidification in axons, become undetectable. To test this possibility, we blocked proteolytic processing using the cysteine protease inhibitor, E64. If a fraction of transported reovirus puncta become non-fluorescent due to conversion to cores, we predict that inhibiting proteolytic processing would lead to an increase in the number of transported fluorescent virions. However, E64 treatment did not affect the number of transported fluorescent reovirus puncta (**Fig 7C**), suggesting that reovirus is transported in neurites prior to disassembly. However, proteolytic processing is required for reovirus infection of neurons, as E64 treatment significantly diminished infection of CNs by virions but not by ISVPs, which bypass the requirement for proteolytic processing (**Fig 7D**) [56].

Proteolytic processing results in the generation of reovirus cores, which can be detected in the soma by 1 h post-adsorption with virions (**Fig 7E and 7F**). Viral replication organelles, identified using antibodies against the viral nonstructural protein σNS also are found concentrated primarily in the DRGN soma, further pinpointing soma as the site of viral replication following disassembly (**Fig 7G**). Together, these results demonstrate that reovirus is transported retrograde in axons as intact virions in non-acidified vesicles, and acidification of vesicles and virion disassembly occur after reaching the neuronal soma, where viral cores enter the cytoplasm and establish replication organelles.

## Discussion

**Fig 8:**
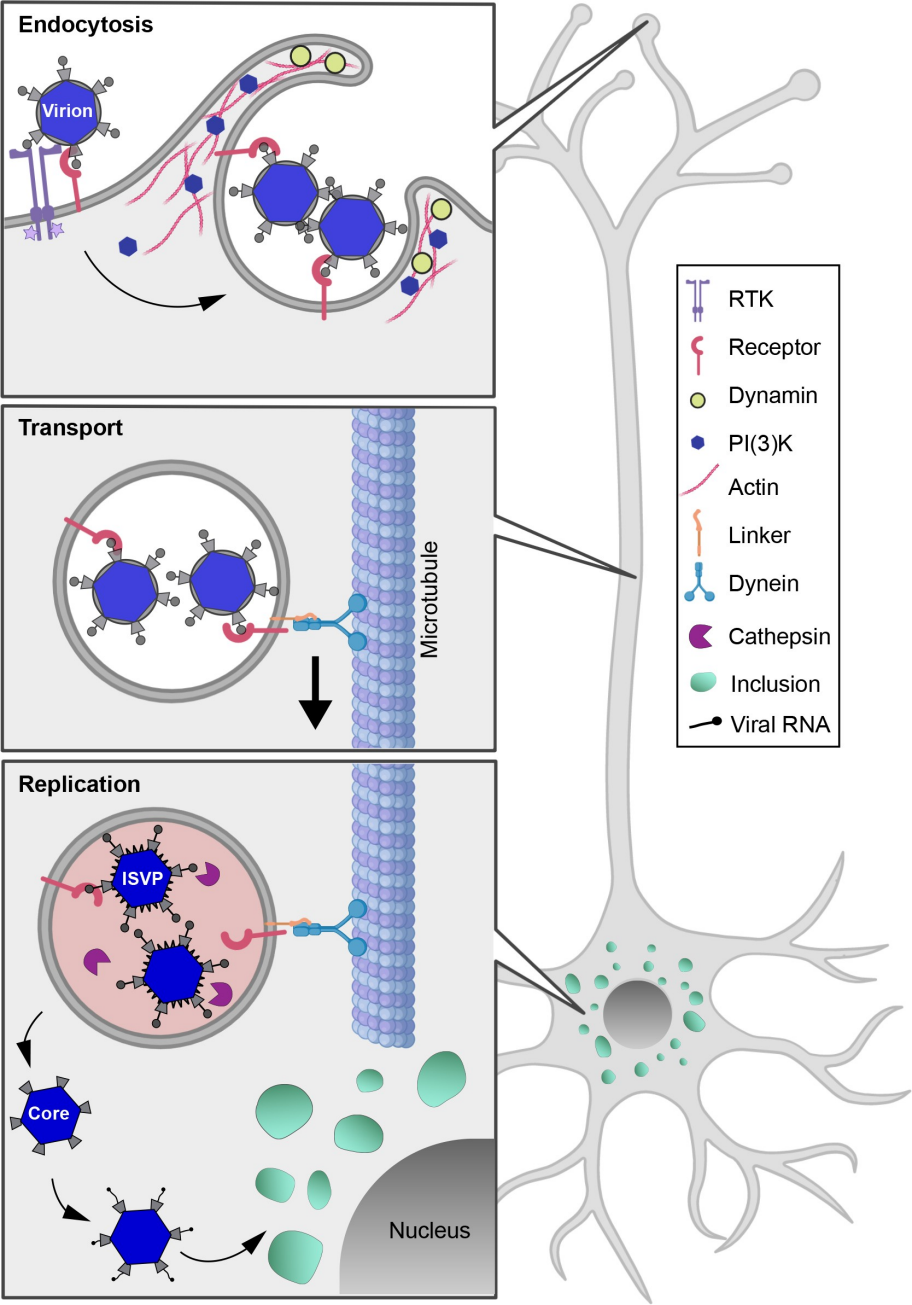
Schematic of reovirus entry and transport in neurons. Reovirus entry in neurons is mediated by macropinocytosis and requires actin, dynamin, PI(3)K, and receptor tyrosine kinase (RTK). Following entry, multiple intact virions are transported together in non-acidified vesicles by dynein motors along microtubule tracks in the retrograde direction. Vesicle acidification in the soma triggers proteolytic disassembly of virions, which enables release of cores into the cytoplasm. Transcription of viral RNAs from cores leads to the establishment of viral replication factories or inclusions.

Studies to understand the cell biology of reovirus infection have relied primarily on non-polarized transformed cell lines. While these studies have provided valuable insights, reovirus infects multiple polarized cells during systemic infection, including intestinal and airway epithelial cells, vascular endothelial cells, and neurons [10, 70, 71]. Examination of reovirus infection of polarized epithelial and endothelial cells has revealed mechanisms of reovirus entry and egress that differ from strategies used in non-polarized cells [5, 28, 72]. Such mechanistic differences demonstrate a need for the use of more representative cell types to understand reovirus infection in a physiological context and, in particular, to study neuronal infection. Here, we established primary neuron cultures from tissues that are infected by reovirus *in vivo* to define early steps in neural infection by reovirus. Using well-characterized, small-molecule inhibitors of endocytosis, we discovered that reovirus is internalized into neurons primarily through macropinocytosis. Following internalization, reovirus undergoes dynein-mediated fast axonal transport in the retrograde direction in vesicles that are predominantly non-acidified. Acid-dependent disassembly of reovirus occurs in the neuronal soma where viral replication organelles are subsequently formed. These findings establish neuron-specific mechanisms used by reovirus to enter and traverse through the highly polarized structure of these cells (**Fig 8**).

Neuronal virus infection often is initiated by invasion of innervating termini at peripheral tissues, followed by retrograde axonal transport and establishment of infection in the soma. While some viruses including alphaherpesviruses and vesicular stomatitis virus have the capacity for anterograde transport and spread after replication [39, 73], others such as measles virus and poliovirus are limited to unidirectional retrograde transport for neural spread [74, 75]. We demonstrate using DRGNs cultivated in microfluidic devices that reovirus readily spreads through retrograde transport but exhibits limited spread in the anterograde direction (**Fig 2D**). Following inoculation of the somal compartment, a majority of the soma became infected, but little virus was detected in the axonal compartment (**Fig 2E**). These results suggest that reovirus progeny produced in the soma do not engage host factors for either anterograde transport or egress from axons. Fluorescently labeled reovirus puncta traffic predominantly in the retrograde direction in DRG axons, with little reversal in direction or anterograde motion, supporting the hypothesis that poor transport may limit anterograde virus release. It also is possible that reovirus undergoes anterograde transport [76, 77], but egress or transmission through axonal termini requires direct synaptic contacts [73, 74, 78], which may be absent between the DRGNs and L929 cells used here. Nevertheless, *in vivo* observations of reovirus dissemination to the CNS through the vagus nerve following intestinal infection [19] or through peripheral motor neurons following inoculation of limb musculature [18, 20, 38] are consistent with the use of predominantly retrograde transport by reovirus.

Neuronal endocytosis is highly regulated, especially at the axonal termini, where endocytosis is required for synaptic vesicle recycling and neuronal activity. Viruses exploit neuronal endocytic machinery for entry following receptor engagement. Dengue virus, orthobunya virus, and rabies virus use clathrin-dependent mechanisms to enter neurons [52, 79, 80], whereas Japanese encephalitis virus uses a clathrin-independent mechanism [81]. We discovered that the major pathway of reovirus internalization in neurons differs from that in non-neuronal cells. Reovirus internalization following JAM-A engagement depends on clathrin and integrin in non-neuronal cells [26, 27, 82]. However, inhibition of CME in neurons did not alter reovirus internalization or transport (**Fig 5C; S4B Fig**), indicating that reovirus uses a clathrin-independent pathway to enter neurons. This is not surprising, as JAM-A is dispensable for neural infection by reovirus [35], and endocytosis following binding to either NgR1 or a yet unknown neural receptor may use a different pathway. In both CNs and DRGNs, reovirus internalization and subsequent transport are sensitive to treatment with amiloride (**Fig 6B; S4B Fig**), and virions are co-transported with a fluorescent fluid-phase marker (**Fig 5D and 5E**). These findings suggest that macropinocytosis is the dominant pathway for reovirus internalization in neurons. Moreover, several host factors that mediate macropinocytosis, including actin, dynamin, and PI3K, are required for reovirus internalization and transport in neurons (**Fig 6D**). Inhibiting macropinocytosis diminishes reovirus infectivity (**Fig 6C**), suggesting that macropinocytosis-mediated entry is essential for productive infection. However, our results do not completely exclude the use of other endocytic mechanisms for reovirus neuronal entry. We found that blocking macropinocytosis does not abolish internalization or trafficking of reovirus (**Fig 6B and 6D; S4B Fig**). While these results are potentially due to incomplete inhibition of macropinocytosis by pharmacological treatments, it also is possible that reovirus uses other clathrin-independent endocytic mechanisms as minor pathways of entry into neurons. Such promiscuity in the use of multiple entry mechanisms also is observed with other viruses including chikungunya virus, herpes simplex virus, and simian virus 40 [83–85]. Nevertheless, our results establish macropinocytosis as a major pathway mediating reovirus entry into neurons. Although multiple viruses from diverse families use macropinocytosis for cell entry, this is an unusual mechanism of virus entry in neurons.

Although fluid uptake during macropinocytosis is non-specific, viruses still must attach to receptors for effective internalization through this mechanism. Accordingly, the capacity of different reovirus serotypes to bind [42] correlates with the capacity for entry and transport in CNs (**Figs 1B, 1D and 3E**). Following cell binding, reovirus may use endogenous macropinocytosis [58–60] or specifically trigger it for neuronal entry. Many viruses, including adenoviruses 3 and 35 [86, 87], human cytomegalovirus [66], influenza A virus [88], Nipah virus [89], and vaccinia virus [90], trigger macropinocytosis through RTKs or integrins after binding to cell-surface moieties. The sensitivity of reovirus entry and transport to a tyrosine kinase inhibitor, genistein (**Fig 6D**), suggests that reovirus activates macropinocytosis in neurons through RTKs. However, genistein also may affect subsequent steps in reovirus entry through macropinocytosis by inhibiting non-RTKs such as src kinases, which have critical functions in the formation and trafficking of macropinosomes [91]. Interestingly, src kinases are essential for productive post-endocytic sorting of reovirus in non-neuronal cells [92]. Such cell type-dependent alternative functions in reovirus endocytosis also may be shared by integrins, which are required for CME of reovirus in non-neuronal cells [26, 82] and may be used to trigger macropinocytosis in neurons. Context-specific use of host factors by viruses can provide plasticity in the use of conserved interactions. Further studies should clarify how reovirus uses or triggers macropinocytosis for neuronal entry and the function of this mechanism in neuropathogenesis.

Alphaherpesviruses enter neurons directly at the termini and recruit motors for retrograde transport, whereas others such as adenovirus, poliovirus, and rabies virus undergo transport within endosomes in axons [44, 45, 52, 75]. Multiple lines of evidence, including cotransport of multiple reovirus particles (**Fig 4**), transport with a fluid-phase marker (**Fig 5D and 5E**), and the presence of reovirus within large vesicles in axons *in vivo* [15], indicate that reovirus is contained within vesicles, presumably macropinosomes, during axonal transport by dynein (**Fig 3A**). Host factors that link reovirus inside the transported vesicle to dynein are not known. Macropinosomes acquire Rab7 as a part of their maturation process [93, 94]. Since Rab7 can recruit dynein via interacting partners [95, 96], it may link reovirus-containing vesicles to dynein. Some neurotropic viruses such as alphaherpesvirus can stimulate synthesis of dynein-regulatory factors to promote their transport following neuronal entry [9]. However, we found that reovirus, similar to rabies virus [97], does not require synthesis of new proteins for axonal transport (**S3 Fig**). During transport in axons, reovirus-containing vesicles remain predominantly non-acidified (**Fig 7A and 7B**). Other cargoes, such as adenovirus, rabies virus, and tetanus toxin, also travel along axons in non-acidic vesicles marked by Rab7 GTPase [40, 44, 52, 98]. These diverse cargoes may share a similar mechanism to prevent endosome acidification during long-distance transport to prevent early entry at sites not suitable for replication. Reovirus-containing vesicles acidify in the soma (**Fig 7A and 7B**), where reovirus cores are subsequently detected (**Fig 7E and 7F**). Such acid-dependent uncoating in the soma also is exhibited by adenovirus and rabies virus [44, 52] and is consistent with the concentration of cathepsin-containing late endosomes in the soma and proximal processes of neurons [99, 100].

Collectively, our study provides insights into the first steps of neural infection by reovirus (**Fig 8**). Importantly, we find that different mechanisms are used by the virus to enter neurons and non-neuronal cells, emphasizing the need to study cell-type-specific mechanisms to understand viral pathogenesis and also to inform engineering of viruses as effective oncolytic agents and gene-delivery vectors. For example, variants of reovirus T3D and adenovirus are in clinical trials for use as oncolytic agents [101, 102]. Both viruses efficiently kill cancer cells [101, 102] and use macropinocytosis for cell entry [103]. Cancer cells also commonly display increased macropinocytosis activity, which is exploited to deliver drug treatments [104, 105]. Further knowledge about the host and viral components required for macropinocytic uptake and the fate of cargoes internalized via this route will enable tailored design of viruses for targeted and combined delivery with cancer therapeutics. Finally, there are striking similarities in mechanisms used for neuronal entry and trafficking by reovirus and misfolded proteins implicated in Alzheimer disease, amyotrophic lateral sclerosis, and Parkinson disease. Protein aggregates implicated in these neurodegenerative diseases enter neurons via macropinocytosis and traffic to propagate between cells in a prion-like fashion [61, 106, 107]. Targeted inhibition of macropinocytosis and transport in neurons through a detailed understanding of these pathways holds promise for therapeutic intervention of neurodegenerative diseases. Our study sets the stage for the use of reovirus as a tool to dissect neurobiology with the potential to enhance oncolytic design and aid in understanding and treatment of neuropathologies.

## Materials and methods

### Cell lines and reovirus stocks

Spinner-adapted L929 cells (obtained from the Bernard Fields laboratory; ATCC CCL-1) were grown in either suspension or monolayers in Joklik's modified Eagle's minimal essential medium (US Biological, M3867) supplemented to contain 5% fetal bovine serum (FBS; VWR, 97068–085), 2 mM L-glutamine, 100 units/ml of penicillin, 100 μg/ml of streptomycin, and 0.25 μg/ml of amphotericin B.

Reovirus strains T3SA+ and T3SA- differ from T1L only in the S1 gene segment that encodes the attachment protein σ1. T3SA+ and T3SA- encode σ1 with primary amino acid sequence of the strain T3C44-MA and T3SA- encodes an additional point mutation (P204L) in σ1 that renders it deficient in sialic acid binding [22]. All three reovirus strains were prepared from laboratory stocks by plaque purification followed by 3–4 passages in L929 cells. Virions were purified from infected L929 cell lysates using a cesium chloride gradient as described [108, 109]. Viral titers were determined by plaque assay using L929 cells and expressed as plaque forming units per ml (PFU/ml) [110]. Particle concentration was determined from the optical density of purified virions at 260 nm using the relationship, 1 OD_260_ = 2.1×10^12^ particles/ml [111]. ISVPs were prepared by treatment of reovirus virions with α-chymotrypsin (0.2 mg/ml, Sigma-Aldrich) at 37°C for 60 min [56].

Reovirus particles were labeled with Alexa Fluor 488 TFP ester or Alexa Fluor 647 succinimidyl ester (Invitrogen) to generate fluorescent virions [69]. For labeling, reovirus particles were diluted to 6×10^12^ particles/ml in freshly prepared 50 mM sodium bicarbonate buffer and incubated with 20 μM dye at room temperature for 90 min, protected from light. Labeled virions were dialyzed at 4°C overnight against phosphate-buffered saline (PBS), exchanging the buffer 2–3 times to remove unreacted dye.

### Ethics statement

All animal work was conducted in accordance with the Public Health Service policy and approved by the University of Pittsburgh Institutional Animal Care and Use Committee (protocol #17101553). All personnel adhered to applicable federal, state, local, and institutional laws and policies governing ethical animal research. This includes the Animal Welfare Act (AWA), the Public Health Service (PHS) Policy, the Principles for the Utilization and Care of Vertebrate Animals Used in Testing, Research and Training, and the Health Research Extension Act of 1985.

### Isolation and culture of primary neurons

Primary neuron cultures were established from Sprague-Dawley rat embryos, E17.5 for CNs and E14.5 for DRGNs. Timed pregnant rats were obtained from Charles Rivers and housed at the University of Pittsburgh facilities until euthanasia by thoracotomy under isoflurane anesthesia. Fetuses were decapitated and stored on ice in Hank’s balanced salt solution without calcium and magnesium (HBSS; Corning) while isolating required tissues.

**CNs:** Cortices were isolated from decapitated fetuses and processed to culture CNs as described [21]. Dishes to culture CNs were coated at 4°C overnight with 10 μg/ml poly-D-lysine hydrobromide (Sigma-Aldrich, P0899) and 1 μg/ml laminin (Corning, 354232) diluted in Neurobasal medium (Gibco, 21103049). CNs were plated at a density of 2×10^5^ viable cells either per dish on the depressed, glass-covered area of 35 mm glass bottom dishes (MatTek, P35G-1.5-14-C) or per well in 24-well plates (Greiner Bio-One, 82050–892). Cultures were maintained in Neurobasal medium supplemented to contain 1x B-27 (Gibco, 17504044), 50 units/ml penicillin, 50 μg/ml streptomycin, and 0.6 mM GlutaMAX (Gibco, 35050079). One-half of the culture medium was changed every 3–4 days for 8–14 days before experimental manipulations.

**DRGNs:** Glass bottom MatTek dishes used to culture DRGNs were plasma-treated (Harrick plasma cleaner, PDC-32G) to improve hydrophilicity of culture surfaces. This treatment was essential to retain adhesion of DRGNs to dishes longer than 5 days in culture. All dishes to culture DRGNs were coated sequentially with 0.5 mg/ml poly-D-lysine hydrobromide and 26 μg/ml laminin at 37°C for 4–16 h.

To isolate DRGs, internal organs were removed from decapitated fetuses, and the spinal cord with attached DRGs was resected from the spinal column. DRGs were isolated and stored in Hibernate-E medium (Gibco, A1247601) up to 10 days before dissociation for culture. Papain (20 units/ml, Worthington) was activated by incubation at 37°C for 20 min in HBSS containing 5.5 mM L-cysteine and 0.2 mg/ml sodium bicarbonate. DRGs were washed with HBSS and treated with the activated papain solution at 37°C for 17 min, followed by incubation in HBSS containing 1 mg/ml ovomucoid protease inhibitor (Worthington, LK003182), 1250 units/ml DNase I (Sigma, D5025), and 1 mg/ml collagenase A (Roche, 10103578001) at 37°C for 10 min. DRGs were washed twice with HBSS and dissociated mechanically by triturating with a fire-polished glass Pasteur pipette. Viable cells were plated at a density of 5–10×10^4^ cells per dish on 35 mm glass bottom MatTek dishes.

DRGN cultures were maintained in a 1:1 mixture of Neurobasal and UltraCULTURE serum-free (Lonza, BE12-725F) media supplemented to contain 1x N-2 (Gibco, 17502048), 1x B-27, 3% Hyclone FBS (GE Healthcare, SH30088.03), 2 mM GlutaMAX, 100 units/ml of penicillin, 100 μg/ml of streptomycin, and 20–50 ng/ml nerve growth factor (NGF; Gibco, 13257019). The day after plating, cultures were treated with 2 μM Cytosine β-D-arabinofuranoside (Sigma-Aldrich, C1768) in complete medium for 48 h to inhibit proliferation of non-neuronal cells in culture. One-half of the culture medium was changed every 3–4 days thereafter for 8–14 days before experimental manipulations.

**DRGN culture in microfluidic devices:** Microfluidic devices with 450 μm long microgrooves preassembled on optically transparent plastic (Xona microfluidics, XC450) were sterilized with 90% ethanol according to manufacturer’s instructions. Devices were subsequently coated sequentially with 0.5 mg/ml poly-D-lysine hydrobromide and 26 μg/ml laminin at 37°C for 4–16 h. Dissociated DRGs, 1–3×10^5^ viable cells, were plated in one compartment of a microfluidic device, designated as the “somal compartment.” To promote extension of axons into the opposing “axonal compartment,” a gradient of NGF was introduced by adding medium containing 20 ng/ml NGF in the somal compartment and 50 ng/ml NGF in the axonal compartment. Neurons were cultivated for 8 days to allow axon penetration into the microgrooves and development of axonal network in the opposing compartment before use in experiments.

### Antibodies

Primary antibodies used for indirect immunofluorescence to stain specific structures include: guinea pig anti-σNS for reovirus inclusions [112], T1L core-specific rabbit antiserum for reovirus cores [113], mouse anti-TUJ1 (BioLegend, 801201) for neurons, chicken anti-MAP2 (Abcam, ab92434) for dendrites, and mouse anti-non-phosphorylated neurofilament H (Millipore Sigma, SMI-32) for axons. Equal volumes of sera from rabbits immunized and boosted with T1L or T3D reovirus strains were mixed [114], adsorbed on rat CN culture to deplete non-specific antibodies, and used to stain reovirus-infected cells. Alexa Fluor conjugated secondary antibodies were used for visualization of antigens by indirect immunofluorescence. Anti-core serum was used at a 1:250 dilution and all other antibodies were used at a 1:1000 dilution.

### Indirect immunofluorescence staining

Cells were fixed using 4% paraformaldehyde and 0.2% glutaraldehyde in PBS at 37°C for 20 min. Fixative was removed and cells were washed three times with PBS and permeabilized and blocked with 0.1% triton X-100 and 5% bovine serum albumin in PBS at room temperature for 1 h. Cells were incubated sequentially with primary and Alexa Fluor-conjugated secondary antibodies diluted in PBS-BGT (PBS containing 0.5% bovine serum albumin, 0.1% glycine, and 0.05% TWEEN 20) at room temperature for 45–60 min and washed three times with PBS-BGT after each incubation. For staining under non-permeabilizing conditions, the same protocol was followed excluding triton X-100 and TWEEN 20 from buffers.

### Treatment with chemical inhibitors

Small molecule inhibitors were reconstituted and stored according to manufacturer’s instructions and used as listed in Table 1.

**Table 1:** Treatment of neurons with small molecule inhibitors.

Small Molecule	Source, Catalog Number	Final Concentration	Duration of Pre-treatment
Ciliobrevin A	Sigma-Aldrich, 250401	50 μM	1 h
Cycloheximide	Sigma-Aldrich, C7698	50 μg/ml	1 h
Dynasore	Abcam, ab120192	150 μM	1 h
E64	Sigma-Aldrich, E3132	200 μM	4 h
5-(N-Ethyl-N-isopropyl)amiloride or EIPA	Sigma-Aldrich, A3085	25 μM	1 h
Genistein	Sigma-Aldrich, G6649	200 μM	1 h
Jasplakinolide	Sigma-Aldrich, J4580	0.5 μM	20 min
Nocodazole	Sigma-Aldrich, M1404	30 μM	1 h
PitStop2	Abcam, ab120687	25 μM	30 min
Wortmannin	Cayman Chemicals, 10010591	200 nM	1 h

List of small molecule inhibitors used in this study along with their sources, final concentrations of use, and durations of pre-treatment of cells.

To study the effects of small molecule inhibitors on reovirus entry, transport, or infectivity, CNs or DRGNs were pre-treated with small molecule inhibitors at 37°C for the indicated time intervals. Pre-treatments were carried out with Ciliobrevin A or PitStop2 diluted in HBSS containing calcium and magnesium and all other drugs diluted in complete culture medium and followed by inoculation with reovirus suspended in culture medium containing drugs.

### Reovirus infection, imaging, and quantification

In experiments comparing effects of small molecule inhibitors on reovirus infectivity, CNs in culture were pre-treated as described above with small molecule inhibitors or a corresponding vehicle control, and then inoculated with T3SA+ virions or ISVPs at a multiplicity of infection (MOI) of 50,000 particles per cell in the presence of inhibitors in Neurobasal medium. In experiments comparing infectivity of reovirus serotypes, CNs or DRGNs in culture were inoculated with reovirus at an MOI of 500 or 50 PFU/cell, respectively, in Neurobasal medium.

Following incubation at 37°C for 1 h, the inoculum was removed, and cells were washed twice with HBSS and incubated in complete culture medium (containing inhibitors, where relevant) at 37°C for 24 h before fixing and immunofluorescence staining. For each condition, 9–16 fields-of-view from duplicate samples were imaged using the LionHeart FX imager. Neurons were identified by TUJ1 or MAP2 staining and infected neurons that were stained by reovirus antiserum were enumerated manually. Infectivity was normalized to controls (not treated with drugs) included in each experiment to account for variations between neuron preparations and number of days in culture.

To infect DRGNs in microfluidic devices, culture medium in either the somal or axonal compartment was replaced with 150 μl inoculum containing T3SA+ virions at an MOI of 500 PFU/cell diluted in complete medium. Fluidic isolation of the inoculum (i.e., restriction of active diffusion of inoculum into the opposing compartment) was achieved by maintaining a 150 μl higher volume in the opposing compartment at all steps. Cells were incubated with reovirus at 37°C for 1 h, washed twice with HBSS, and returned to complete medium. Devices were incubated at 37°C and media from both compartments were collected at 24 h intervals and replaced with fresh medium. Cells were fixed at 72 h post-inoculation and neurons and reovirus antigens were visualized by indirect immunofluorescence. Viral titers in collected supernatants were determined by plaque assay.

### Live confocal microscopy of reovirus transport

Neurons plated on glass bottom dishes were treated with small molecule inhibitors or vehicle controls and subsequently incubated with 3×10^10^ (for CNs) or 1×10^9^ (for DRGNs) particles of fluorescently-labeled reovirus diluted in the corresponding complete medium containing inhibitors at 37°C for 30 min. Inoculum was removed, cells were washed twice with HBSS, and complete medium containing inhibitors was added. Reovirus transport in neurons was imaged using a Leica TCS-SP8 laser scanning confocal microscope equipped with an environmental chamber (GSI TOKAI HIT Standard Heating Stage Top Incubator) and a lens heater, which allow maintenance of cells at 37°C for live imaging. Dishes were allowed to equilibrate to 37°C in the heated chamber for 10 min before imaging. Bright field images constructed from PMT signals were used to select random 82 x 82 μm regions uniformly covered with soma and neurites. Time-lapse images of a single optical section of cells (pinhole = 1 AU) were acquired using a 63x objective (NA = 1.4; HC PL APO) and a HyD detector with an optical zoom of 2.25 and a frame interval of 0.26 s. At least 10 fields-of-view spread throughout each dish were imaged with imaging time limited to 30 min per dish.

To monitor directional trafficking of reovirus, the axonal compartment of microfluidic devices with DRGN cultures was treated with 3×10^10^ particles of fluorescently-labeled virions at 37°C for 45 min. Inoculum was removed, cells were washed twice with HBSS, and complete medium was added maintaining fluidic isolation of the inoculated chamber. Time-lapse images of reovirus transport through microgrooves, each containing multiple axons, were acquired under live-cell imaging conditions described above. At least 20 grooves were imaged per dish with a 0.26 s interval between frames and imaging time per groove was limited to 60 s.

### Analysis of reovirus transport

**Number of particles trafficking per field:** Fiji ImageJ was used to project maximal intensities from time-lapse images of reovirus transport in neurons into a single plane. Trajectories of reovirus particles were identified from the projected images and the number of particles trafficking along each trajectory was manually enumerated from the time-lapse images. Total number of particles trafficking per field under each condition was normalized to corresponding control samples from the same experimental day.

**Transport kinetics:** A custom python script was used to execute the TrackMate (v3.7.0) plugin for Fiji ImageJ for automated tracking of fluorescent reovirus particles in time-lapse images and to obtain corresponding trajectory information. A median filter (3x3 pixels) was applied to all images. Puncta with an initial estimated “blob diameter” of 0.375 pixels (1 pixel = 0.16 μm under imaging setup) were detected using the difference of Gaussian (DoG) filter and puncta with a manually-determined intensity threshold were considered for further analysis. The simple Linear Assignment Problem (LAP) tracker was used to build trajectories with a maximum linking distance of 2.5 μm, and a maximum distance of 3 μm and a frame interval of 3 for gap-closing. Only tracks with displacement greater than 6.5 μm were considered for further analysis.

Parameters describing transport dynamics were computed using a custom MATLAB script. Instantaneous velocities were calculated as the displacement of puncta in consecutive ~ 1 s intervals (4 frames). Particles were defined as “in motion” if instantaneous velocities were > 0.15 μm/s and “paused” if < 0.15 μm/s. Pause duration was defined as the cumulative time spent by a punctum in the paused state in consecutive 1 s intervals. Track speed was calculated as the distance traveled by each punctum divided by its tracked duration.

### Transferrin uptake quantification

To determine the PitStop2 concentration for efficient inhibition of CME, CNs were treated with a range of concentrations of PitStop2 (0 to 30 μM) diluted in HBSS with calcium and magnesium for 30 min. Cells were subsequently incubated with 25 μg/mL Alexa Fluor 488-labeled transferrin (Invitrogen, T13342) in the presence of PitStop2 at 37°C for 20 min. Cells were then washed twice with stripping buffer (10 mM glycine, 150 mM NaCl, pH 2.5) to remove surface-bound transferrin and fixed using 4% paraformaldehyde. Transferrin uptake was quantified under each condition as the integrated fluorescence intensity within a 2.8 x 2.8 μm area of soma. Results were averaged from three experimental repeats, each including duplicate samples, and at least 50 soma from each sample.

### Quantification of internalized reovirus

CNs cultured in glass bottom dishes were treated with small molecule inhibitors of endocytosis or vehicle controls under aforementioned conditions (Table 1). Subsequently, cells were inoculated with 3×10^10^ particles/dish of Alexa Fluor 647-labeled T3SA+ (red) suspended in complete medium containing inhibitors at 37°C for 40 min. A vehicle-treated sample inoculated at 4°C was used as a control for complete inhibition of endocytosis. Inoculum was removed, and cells were washed twice with HBSS, fixed, and incubated under non-permeabilizing conditions with rabbit anti-reovirus polyclonal serum and Alexa Fluor 488-labeled anti-rabbit IgG (green) for visualization of extracellular reovirus by indirect immunofluorescence. Single optical sections of at least 10 fields-of-view (82 x 82 μm) uniformly covered with neurites were imaged per dish. Images were processed and analyzed using Fiji ImageJ. Positions of red-fluorescent reovirus puncta (total virus) were identified using the “find maxima” function and the intensity of the green channel (extracellular virus stained by antibody) at the corresponding positions was recorded. Reovirus puncta were categorized as internalized if the green channel intensity was comparable to the background.

### Analysis of reovirus co-transport with transferrin and dextran

Alexa Fluor 647-conjugated transferrin (Invitrogen, T23366) was used to track CME and Alexa Fluor 647-conjugated 10,000 MW dextran (Invitrogen, D22914) was used as a marker for fluid-phase uptake during macropinocytosis. To study reovirus co-transport with cargoes taken up by distinct endocytic pathways, CNs were incubated with 3×10^10^ particles of Alexa Fluor 488-labeled T3SA+ reovirus and fluorescent transferrin or dextran at final concentrations of 50 μg/ml and 100 μg/ml, respectively, in culture medium. Cells were incubated at 37°C for 30 min, washed twice with HBSS, returned to culture medium, and imaged immediately under live-cell imaging conditions. Multichannel time-lapse images were acquired simultaneously using HyD detectors. Maximal intensity projections of time-lapse images were used to visualize trajectories of transported reovirus particles. Kymographs were generated along individual trajectories using Fiji ImageJ and cotransport of reovirus with transferrin or dextran was determined from the overlap of corresponding fluorescence signals on kymographs.

### Quantification of reovirus colocalization with acidified vesicles

Axonal compartment of microfluidic devices with DRGNs were treated with 3×10^10^ particles of Alexa Fluor 488-labeled T3SA+ virions in complete medium while maintaining fluidic isolation at 37°C for 30 min. Inoculum was removed, the axonal compartment was washed twice with HBSS, and the cells were incubated in complete medium at 37°C for 1.5–3.5 h. Before imaging, both somal and axonal compartments were treated with 150 nM LysoTracker Deep Red (Invitrogen, L12492) in complete medium at 37°C for 20 min to label acidic compartments. Cells were washed twice with HBSS, returned to complete medium, and imaged under live-cell imaging conditions.

Two-channel time-lapse images were acquired first in the microgroove area to capture motions of reovirus and LysoTracker in axons, and then single snapshots were acquired in the somal compartment to capture distribution of fluorescent markers in the neuronal cell bodies. Reovirus co-trafficking with LysoTracker in axons was inferred from kymographs as described above for analysis of co-transport with dextran and transferrin. To analyze reovirus colocalization with LysoTracker-stained vesicles (red puncta) in soma, background was subtracted from the red channel using a rolling ball radius of 5 for all images. Regions of interest containing reovirus puncta (green) were identified using the “Analyze Particles” function and the intensity of red channel at the corresponding regions was recorded. If the red channel intensity was above background, reovirus punctum was considered to be colocalized with LysoTracker.

### Quantification of reovirus cores

DRGNs cultivated in glass bottom dishes were adsorbed with 3×10^10^ particles/dish of Alexa Fluor 647-labeled T3SA+ for 30 min. The inoculum was removed and cells were washed twice with HBSS and incubated at 37°C for 1–3 h before fixation. Cells were subsequently permeabilized and stained with core-specific rabbit polyclonal serum and Alexa Fluor 488-labeled anti-rabbit IgG. Fluorescence images of single optical sections were acquired through the middle of soma from multiple fields-of-view. The soma were outlined using bright field images and only soma containing internalized reovirus particles as identified from Alexa Fluor 647 signal were considered for further analysis. Fluorescent reovirus core puncta within selected soma were identified using the “Find Maxima” function in Fiji ImageJ and enumerated.

### Statistical analysis

All data were analyzed using Graphpad Prism 7. Figure legends specify the number of experimental repeats and the statistical test applied for each analysis. Differences were considered statistically significant when *P*-values were less than 0.05.

## Supporting information

S1 Fig Replication of reovirus following inoculation of DRGs in microfluidic devices. DRGs cultivated in microfluidic devices were adsorbed with T3SA+ virions in the axonal (A) or somal (B) compartment as indicated in the schematics. Viral titers in culture supernatants in the inoculated compartments of microfluidic devices were determined at the times shown. Corresponding titers in the opposing compartments are shown in Fig 2D. Bars indicate means, and error bars indicate SEM. Individual data points represent titers from three independent experiments using a total of five devices. Dashed lines mark the limit of detection.
(TIF)

S2 Fig Dynamics of reovirus transport in neurons. CNs or DRGNs cultivated in glass-bottomed dishes or microfluidic devices (data labeled with MF) were adsorbed with the fluorescently-labeled reovirus strains shown for 30 min. The inoculum was removed, and time-lapse fluorescence images were acquired for 1 min each from 8 to 12 fields-of-view per sample. (A) Representative micrographs show time-lapse images projected onto one plane from a 1-min movie of fluorescently-labeled T3SA+ transport in CNs. Scale bar, 10 μm. (B-D) Trajectories of individual puncta that travel at least 6.5 μm during the imaging interval were analyzed for the following motion characteristics: distribution of instantaneous velocities (B), duration of pauses (C), and the percentage of tracked time spent in the paused state (D). In C and D, bars indicate medians, and error bars indicate 95% confidence intervals from trajectories of *n* > 300 puncta.
(TIF)

S3 Fig Axonal transport of reovirus does not require new protein synthesis. (A) CNs were treated DMSO control or cycloheximide (Table 1), adsorbed with fluorescently-labeled T3SA+ virions for 30 min, and T3SA+ puncta transported per field-of-view were enumerated following treatment. Bars indicate means normalized to untreated controls. (B) CNs treated with cycloheximide were adsorbed with T3SA+ virions at an MOI of 500 PFU/cell, and the number of cells stained with viral antigen per field-of-view was quantified at 24 h post-adsorption. In A and B, bars indicate means and error bars indicate SEM. Data are representative of at least two independent experiments each with two samples. Individual data points are averages from 8 to 12 fields-of-view per sample.
(TIF)

S4 Fig Macropinocytosis and not clathrin-mediated endocytosis is required for reovirus neuronal entry. (A-B) CNs were treated with the drugs shown (Table 1) and adsorbed with Alexa Fluor 647-labeled T3SA+ virions at 4°C (as a negative control for internalization) or 37°C for 40 min. The inoculum was removed, cells were fixed, and internalized virions were identified using indirect immunofluorescence staining under non-permeabilizing conditions. Schematic of strategy (A, left) and a representative micrograph (A, right) with extracellular (yellow) and internalized (red) T3SA+ virions are shown. Scale bar, 5 μm. The number of internalized reovirus puncta per field-of-view was quantified and normalized to DMSO-treated controls (B). (C) CNs were treated with the drugs shown (Table 1). Uptake of transferrin-Alexa Fluor 488 following treatment was quantified and normalized to DMSO-treated controls. In B and C, bars indicate means from two-to-three independent experiments, each with duplicate samples. Error bars indicate SEM. Individual data points represent averages from 8 to 12 fields-of-view per sample. Values that differ significantly from control by ANOVA and Dunnett's test are indicated (*, P < 0.05; **, P < 0.01; ***, P < 0.001; ****, P < 0.0001). (D) CNs treated with EIPA were incubated with Alexa Fluor 488-labeled cholera toxin subunit B (ctxB) at a concentration of 3.3 μg/ml for 30 min. Cells were washed twice with stripping buffer to remove surface-bound toxin, fixed using 4% paraformaldehyde, and the mean fluorescence intensity of toxin internalized into neuronal cell bodies was quantified. Bars indicate means from three independent experiments, each with duplicate or triplicate samples. Error bars indicate SEM. Individual data points represent averages from 15 to 20 cells per sample. (E) DRGNs were treated with EIPA and adsorbed with T3SA+ virions at an MOI of 5 PFU/cell. Infectivity was quantified 24 h post-adsorption. Bars indicate the mean number of infected neurons per 776 x 776 μm field. Error bars indicate SEM. Data are representative of three independent experiments, each with one or two samples. Individual data points are averages from 10 fields-of-view per sample. **, *P* < 0.01 determined by *t*-test.
(TIF)

S1 Movie Reovirus is actively transported in CNs. CNs (bright field) cultivated on glass bottom dishes were incubated with fluorescent T3SA+ virions (green) for 30 min. Yellow lines track viral puncta (identified by magenta circles) transported longer than 6.5 μm during the 1 min imaging interval.
(AVI)

S2 Movie Reovirus traffics retrograde in DRGNs. Fluorescent T3SA+ virions (green puncta) applied to the axonal compartment traffic retrograde towards the soma in DRGN axons within the grooves (bright field) of the microfluidic device. The device is oriented in the images with somal compartment to the left and axonal compartment to the right of the grooves.
(WMV)

S3 Movie Multiple reovirus particles can be transported together in axons. CNs were adsorbed with a 1:1 mixture of Alexa Fluor 488- (green) or Alexa Fluor 647-labeled (red) T3SA+ virions. Multiple puncta containing both fluorophores (tracked using yellow circles) can be observed to traffic in time-lapse images.
(WMV)

S4 Movie Reovirus co-traffics in axons with a marker for macropinocytosis. CNs were incubated with Alexa Fluor 488-labeled T3SA+ virions (green) and fluorescent 10 kDa dextran (red). Two-color time-lapse images show co-transport of reovirus puncta with dextran (tracked using yellow circles).
(AVI)

S5 Movie Reovirus traffics predominantly in non-acidified vesicles in DRG axons. DRG neurons cultivated in microfluidic device were inoculated in the axonal compartment with Alexa Fluor 488-labeled T3SA+ virions (green) and stained for acidic vesicles using LysoTracker (red). Time-lapse images show transport of reovirus and acidic organelles in axons within a single groove of the device. The device is oriented in the images with somal compartment to the left and axonal compartment to the right of the groove. A majority of reovirus traffics retrograde in non-acidified vesicles that are not stained by LysoTracker. White circles track a few transported reovirus puncta stained by LysoTracker (yellow puncta).
(WMV)

S6 Movie Reovirus colocalizes with acidified vesicles in the DRG soma. DRG neurons cultivated in microfluidic device were inoculated in the axonal compartment with Alexa Fluor 488-labeled T3SA+ virions (green) and stained for acidic vesicles using LysoTracker (red). Time-lapse images show distribution of reovirus and acidic organelles within a single DRGN soma (in the somal compartment) at 3.5 h post-inoculation. Many reovirus puncta within the soma are contained in acidified vesicles stained by LysoTracker (yellow puncta).
(WMV)

S1 Dataset Compilation of raw data used in the manuscript. (XLSX)
