# Supplementary figures and images for: Reovirus uses macropinocytosis-mediated entry and fast axonal transport to infect neurons
Source: PLoS Pathog. 2020 Feb 28;16(2):e1008380. doi: 10.1371/journal.ppat.1008380 (PMC7065821; doi:10.1371/journal.ppat.1008380)

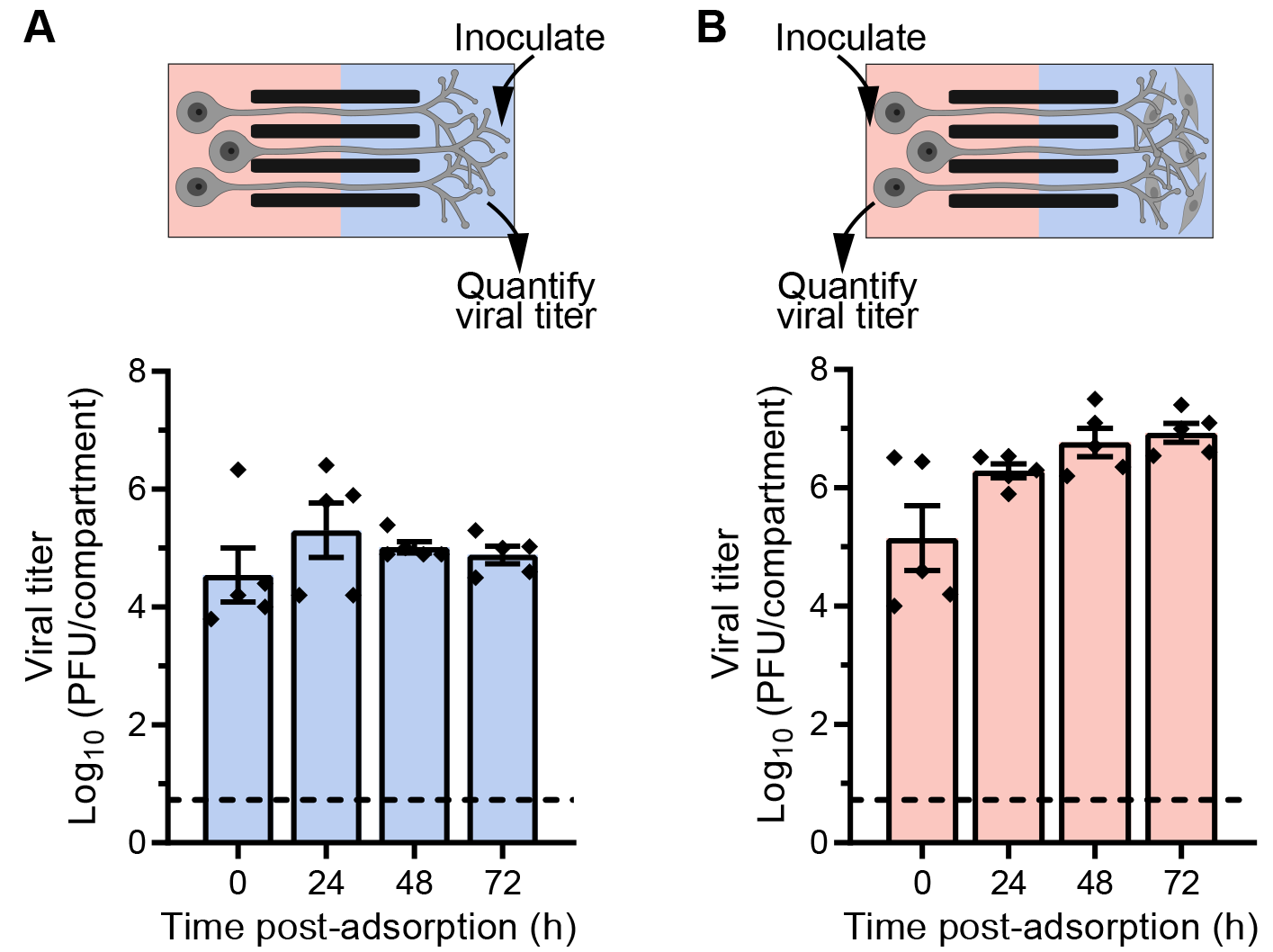

Supplement: S1 Fig — DRGs cultivated in microfluidic devices were adsorbed with T3SA+ virions in the axonal (A) or somal (B) compartment as indicated in the schematics. Viral titers in culture supernatants in the inoculated compartments of microfluidic devices were determined at the times shown. Corresponding titers in the opposing compartments are shown in Fig 2D. Bars indicate means, and error bars indicate SEM. Individual data points represent titers from three independent experiments using a total of five devices. Dashed lines mark the limit of detection. (TIF) [file ppat.1008380.s001.tif]

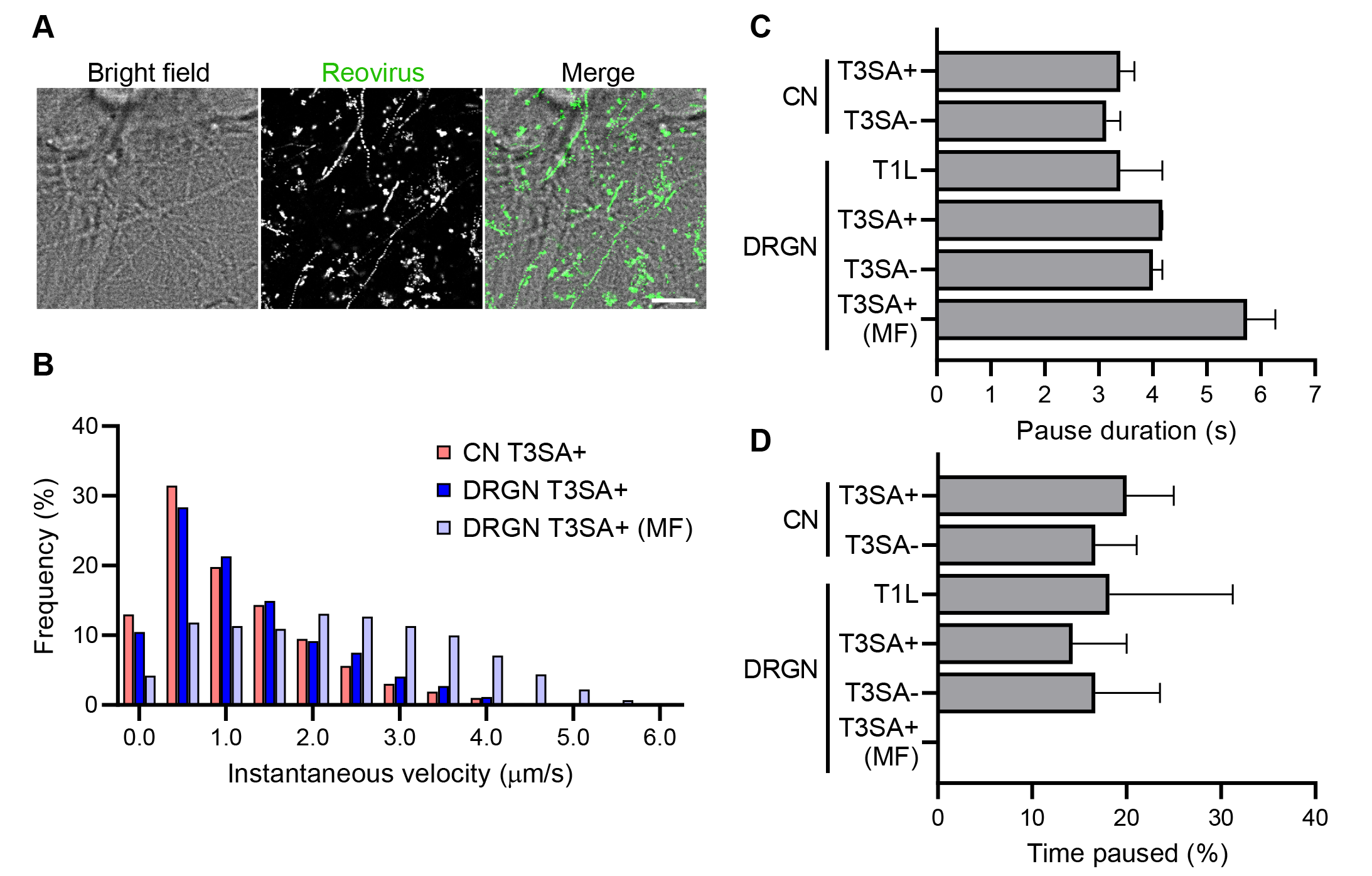

Supplement: S2 Fig — CNs or DRGNs cultivated in glass-bottomed dishes or microfluidic devices (data labeled with MF) were adsorbed with the fluorescently-labeled reovirus strains shown for 30 min. The inoculum was removed, and time-lapse fluorescence images were acquired for 1 min each from 8 to 12 fields-of-view per sample. (A) Representative micrographs show time-lapse images projected onto one plane from a 1-min movie of fluorescently-labeled T3SA+ transport in CNs. Scale bar, 10 μm. (B-D) Trajectories of individual puncta that travel at least 6.5 μm during the imaging interval were analyzed for the following motion characteristics: distribution of instantaneous velocities (B), duration of pauses (C), and the percentage of tracked time spent in the paused state (D). In C and D, bars indicate medians, and error bars indicate 95% confidence intervals from trajectories of n > 300 puncta. (TIF) [file ppat.1008380.s002.tif]

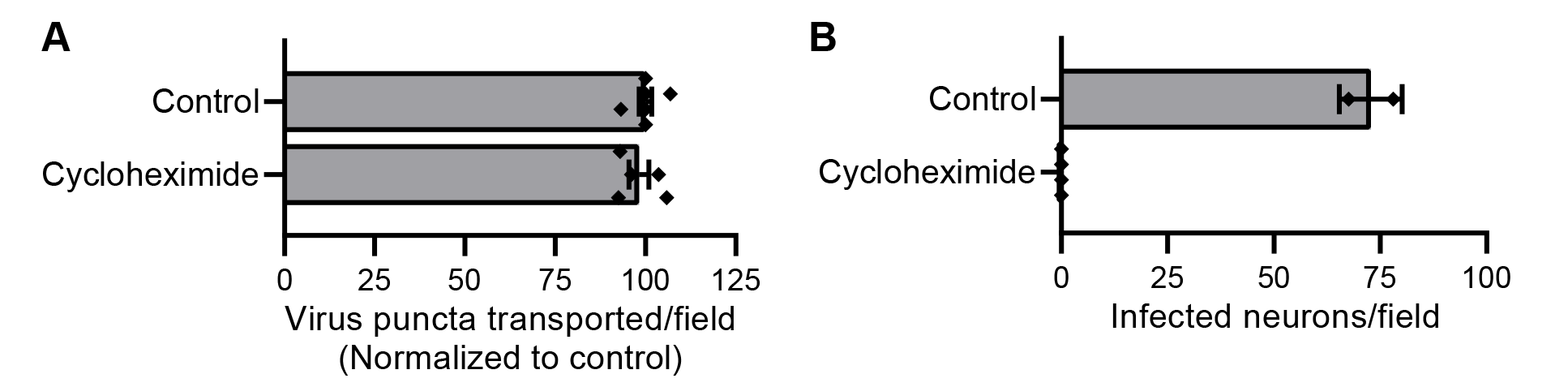

Supplement: S3 Fig — (A) CNs were treated DMSO control or cycloheximide (Table 1), adsorbed with fluorescently-labeled T3SA+ virions for 30 min, and T3SA+ puncta transported per field-of-view were enumerated following treatment. Bars indicate means normalized to untreated controls. (B) CNs treated with cycloheximide were adsorbed with T3SA+ virions at an MOI of 500 PFU/cell, and the number of cells stained with viral antigen per field-of-view was quantified at 24 h post-adsorption. In A and B, bars indicate means and error bars indicate SEM. Data are representative of at least two independent experiments each with two samples. Individual data points are averages from 8 to 12 fields-of-view per sample. (TIF) [file ppat.1008380.s003.tif]

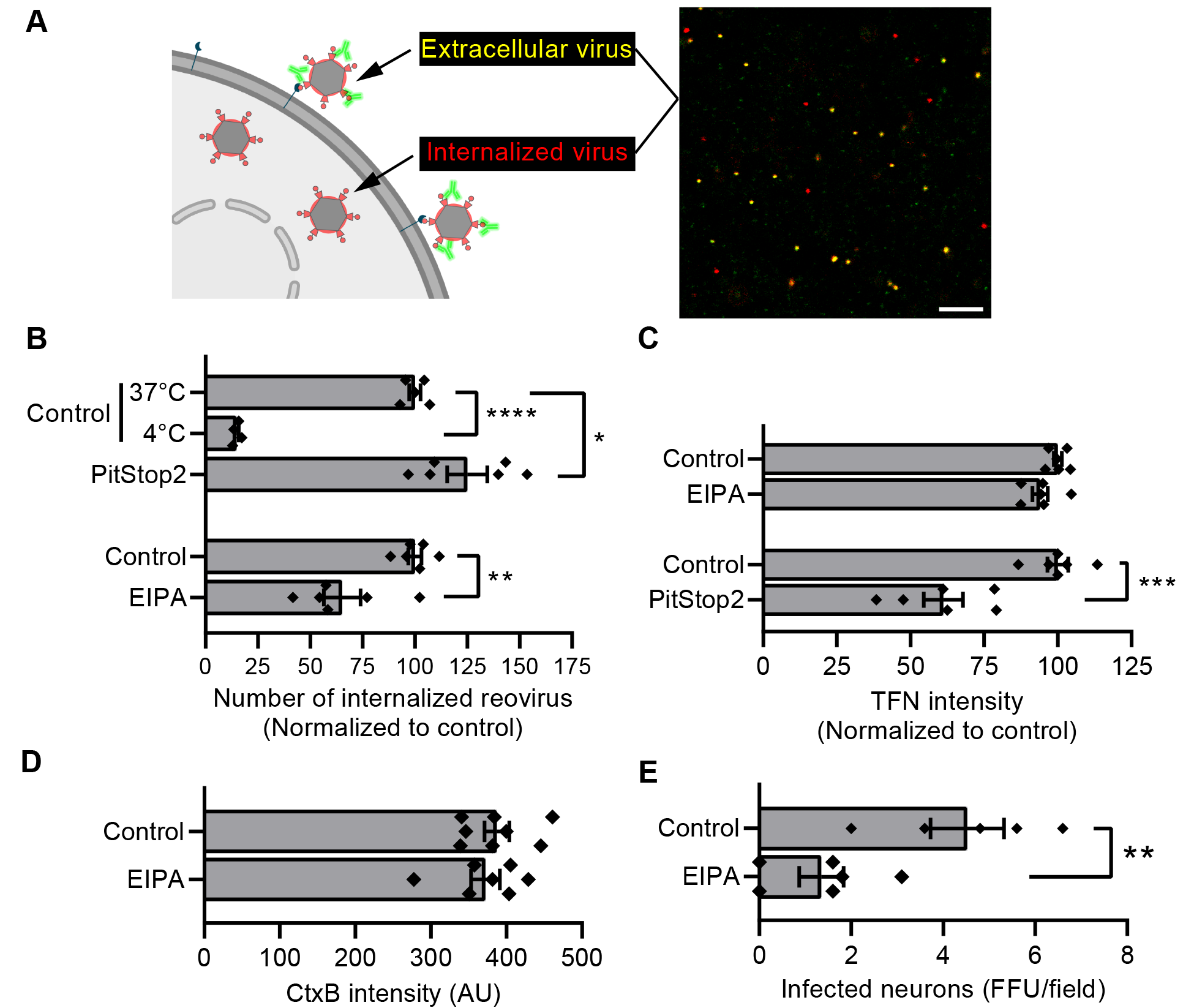

Supplement: S4 Fig — (A-B) CNs were treated with the drugs shown (Table 1) and adsorbed with Alexa Fluor 647-labeled T3SA+ virions at 4°C (as a negative control for internalization) or 37°C for 40 min. The inoculum was removed, cells were fixed, and internalized virions were identified using indirect immunofluorescence staining under non-permeabilizing conditions. Schematic of strategy (A, left) and a representative micrograph (A, right) with extracellular (yellow) and internalized (red) T3SA+ virions are shown. Scale bar, 5 μm. The number of internalized reovirus puncta per field-of-view was quantified and normalized to DMSO-treated controls (B). (C) CNs were treated with the drugs shown (Table 1). Uptake of transferrin-Alexa Fluor 488 following treatment was quantified and normalized to DMSO-treated controls. In B and C, bars indicate means from two-to-three independent experiments, each with duplicate samples. Error bars indicate SEM. Individual data points represent averages from 8 to 12 fields-of-view per sample. Values that differ significantly from control by ANOVA and Dunnett's test are indicated (*, P < 0.05; **, P < 0.01; ***, P < 0.001; ****, P < 0.0001). (D) CNs treated with EIPA were incubated with Alexa Fluor 488-labeled cholera toxin subunit B (ctxB) at a concentration of 3.3 μg/ml for 30 min. Cells were washed twice with stripping buffer to remove surface-bound toxin, fixed using 4% paraformaldehyde, and the mean fluorescence intensity of toxin internalized into neuronal cell bodies was quantified. Bars indicate means from three independent experiments, each with duplicate or triplicate samples. Error bars indicate SEM. Individual data points represent averages from 15 to 20 cells per sample. (E) DRGNs were treated with EIPA and adsorbed with T3SA+ virions at an MOI of 5 PFU/cell. Infectivity was quantified 24 h post-adsorption. Bars indicate the mean number of infected neurons per 776 x 776 μm field. Error bars indicate SEM. Data are representative of three independent [file ppat.1008380.s004.tif]
